# Diagnosis of thyroid neoplasm using support vector machine algorithms based on platelet RNA-seq

**DOI:** 10.1007/s12020-020-02523-x

**Published:** 2020-11-12

**Authors:** Yuling Shen, Yi Lai, Dong Xu, Le Xu, Lin Song, Jiaqing Zhou, Chengwen Song, Jiadong Wang

**Affiliations:** 1 Department of Head and Neck Surgery, Renji Hospital, School of Medicine, Shanghai Jiaotong University, 160 Pujian Road, Pudong District, Shanghai, 200127 China; 2 Fun-med Pharmaceutical Technology (Shanghai) Co., Ltd., RM. A310, 115 Xinjunhuan Road, Minhang District, Shanghai, 201100 China

**Keywords:** Thyroid neoplasm, SVM algorithm, Platelet RNA-seq, Bioinformatics analysis

## Abstract

**Objective:**

To assess the capacity of support vector machine (SVM) algorithms that are developed based on platelet RNA-seq data in identifying thyroid neoplasm patients and differentiating patients with thyroid adenomas, papillary thyroid cancer and metastasized papillary thyroid cancer.

**Methods:**

Platelets were collected and isolated from 109 patients and 63 healthy controls. RNA-seq was performed to find transcripts with differential levels. Genes corresponding to these altered transcripts were identified using R packages. All samples were subsampled into a training set and a validation set. Two SVM algorithms were developed and trained with the training set, using the genes with differential transcript levels (GDTLs) as classifiers, and validated with the validation set. GO and KEGG pathway enrichment analysis were performed using the R package clusterProfiler.

**Results:**

We detected 765 GDTLs (442 up-regulated and 323 down-regulated) in platelets of patients and healthy controls. The algorithm identifying thyroid neoplasm patients achieved an accuracy of 97%, with an AUC (area under curve) of 0.998. The other algorithm differentiating patients with multiclass thyroid neoplasms had an average accuracy of 80.5%. GO analysis showed that GDTLs were strongly involved in biological processes such as neutrophil degranulation, neutrophil activation, autophagy and regulation of multi-organism process. KEGG pathway enrichment analysis revealed that GDTLs were mainly enriched in NOD-like receptor signaling pathway and pathways in endocytosis, osteoclast differentiation, human cytomegalovirus infection and tuberculosis.

**Conclusion:**

Our results indicated that the combination of SVM algorithms and platelet RNA-seq data allowed for thyroid neoplasm diagnostics and multiclass thyroid neoplasm classification.

## Introduction

Thyroid cancer is the most common endocrine cancer, with an increasing incidence of about two fold in the last 25 years and accounting for 2% of all cancers [1]. This overall incidence growth is driven by the widespread use of sensitive imaging techniques [2, 3]. The growth in incidence without corresponding rise in mortality reflects cancer overdiagnosis. Because surgery is the key therapy for most thyroid cancers [4], overdiagnosis usually leads to overtreatment, resulting in the performance of unnecessary thyroidectomies or radioiodine treatments with potential harm to the patient [5]. Therefore, development of an artificial intelligence algorithm with high sensitivity and specificity could help clinicians to diagnose thyroid neoplasm and differentiate thyroid adenomas and carcinomas, thus avoiding overtreatment and also unnecessary fine-needle aspiration biopsy.

It has been reported that blood platelets respond to changes in the host systemic environment during tumorigenesis and cancer metastasis [6], thus resulting in altered platelet behavior [7, 8]. Best et al. [9]. demonstrated that platelet transcriptome could function as a biomarker trove to detect and classify cancer via SVM algorithms. Herein, we aimed to evaluate the capacity of SVM algorithms in identifying patients with thyroid neoplasms and differentiating healthy individuals and patients with thyroid adenomas (TA), papillary thyroid cancer (PTC) and metastasized papillary thyroid cancer (MPTC) based on altered transcript profiles in platelets.

## Materials and methods

### Clinical sample collection

Healthy individuals and patients with biopsy-confirmed TA, PTC and MPTC were recruited from Renji Hospital, Shanghai, China. MPTC is defined as PTC that spreads to lymph nodes. Healthy individuals are voluntary blood donors who undertook thyroid ultrasound to rule out thyroid diseases. This experiment was approved by the Ethical Committee of the Renji Hospital. Written informed consent was obtained from all subjects. Patients with thyroid nodules and following complications were excluded: diabetes, hypertension, cardiovascular diseases, pregnancy and active infection. 2 ml peripheral whole blood samples were collected from all subjects and stored in EDTA-containing tubes. A patient class and a control class were established to classify samples.

### Platelet isolation and RNA extraction

Platelets were isolated from the samples within 24 h after collection to minimize loss of platelet RNA quality and quantity. To isolate platelets, platelet rich plasma (PRP) was separated by a 20-min 1000 rpm centrifugation step at 4 °C. To reduce the risk of contamination of platelet preparation with hemocytes, only 9/10th PRP was drawn and transferred to 1.5 ml Eppendorf tubes, after which platelets were pelleted by a 20-min 3000 rpm centrifugation step. The supernatant was subsequently removed, and platelets were observed as white precipitate. Phosphate buffered saline (PBS) solution was used to wash the platelets and then removed after a 15-min 3000 rpm centrifugation step, after which an instantaneous centrifugation was performed, and a 10-μl pipette was used to remove residual PBS solution. Precipitated platelets were carefully resuspended in RNAlater (Thermo Scientific) and frozen at −20 °C. Contamination of hemocytes in platelet preparations was quantified by a hematology analyzer.

### Library construction and mRNA sequencing

Total platelet RNA of each sample was extracted using RNeasy Micro Kit (Qlagen, cat. no. 74004) per the manufacturer’s instructions. The total RNA was quantified and qualified by Agilent 2100 Bioanalyzer, NanoDrop (Thermo Scientific) and 1% agrose gel. Reverse transcription was achieved with 1 ng of total RNA, oligo (dT) primer and Superscript II reverse transcriptase. To have sufficient platelet cDNA for robust RNA-seq library preparation, each sample was subjected to 20-cycle enrichment PCR, using the KAPA HiFi HotStart ReadyMix PCR kit (Kapa Biosystems, USA), yielding ~200 ng of cDNA. AMPure XP beads were used to purify double-stranded cDNA, which was then treated with End Prep Enzyme Mix to repair both ends and add a dA-tailing in one reaction, followed by a T-A ligation to add adapters to both ends. Size selection of Adapter-ligated DNA was then performed using beads, and fragments of ~420 bp (with the approximate insert size of 300 bp) were recovered. Each sample was then amplified by PCR for 13 cycles using P5 and P7 primers, with both primers carrying sequences which can anneal with flow cell to perform bridge PCR and P7 primer carrying a six-base index allowing for multiplexing. The PCR products were cleaned up using beads, validated using an Qsep100 (Bioptic, China), and quantified by Qubit 3.0 Fluorometer (Invitrogen, USA).

Libraries with different indices were multiplexed and loaded on an Illumina HiSeq instrument per the manufacturer’s instructions (Illumina, USA). Sequencing was carried out using a 2 × 150 bp paired-end (PE) configuration; image analysis and base calling were conducted by the HiSeq Control Software (HCS) + OLB + GAPipeline-1.6 (Illumina) on the HiSeq instrument. The sequences were processed and analyzed by Fengneng Pharmaceutical Technology (Shanghai) Co., Ltd.

### Data analysis and GDTL identification

In order to purify raw data of fastq format, Cutadapt (v1.9.1) was used to remove adapters and ploymerase chain reaction (PCR) primers, and FASTP was used to filter reads with the following standards: (1) reads shorter than 50 bp were filtered; (2) reads with more than 5N bases were filtered; (3) reads with an average quality per base below Q15 were removed. For quality checking, a sample will be excluded if its clean reads account for <80% of its raw reads or its clean reads do not meet quality standards (Q20 > 90% and Q30 > 80%). Clean reads were aligned to human reference genome GRCh38 (ftp://ftp.ensembl.org/pub/release-91/fasta/homo_sapiens/dna/) via STAR (v2.4.2a), yielding BAM files, which were subsequently processed by HTSeq (ve0.6.1) to estimate differential transcript levels of genes. Here, STAR was also used to remove multi-mapping and unmapped reads, and a sample will be excluded if its uniquely mapped reads cannot account for >85% of its total reads before removal. Gene-level differential analysis at transcript-level resolution was performed using Bioconductor package DESeq2. Basemean > 100, |log2(FC)| > 1 and Padj < 0.05 were set to detect GDTLs. VENNY 2.1 (https://bioinfogp.cnb.csic.es/tools/venny/) was used to screen class-specific GDTLs as classifiers for the multiclass algorithm.

### GO and KEGG enrichment analysis

Gene Ontology (GO) is a structured, controlled vocabulary for the classification of gene function at the molecular and cellular level. Kyoto Encyclopedia of Genes and Genomes (KEGG) is a collection of databases that can help to understand large-scale datasets generated by transcriptome sequencing. We performed GO and KEGG pathway enrichment analysis to functionally cluster GDTLs via the R package clusterProfiler.

### Algorithm development

Prior to algorithm development, a train_test_split function implemented in Python 3.7.2 was used to randomly split samples into a training set and a validation set. The ratio of training:validation was set to be 60:40. Each time we executed the algorithms, samples in each sample class were extracted realtimely per this ratio according the total size of their classes to fill the training and validation sets, thus SVM outcomes were dynamic. To test the stability of performances of both algorithms, we execute both algorithms 1000 times and calculated their average accuracies. We also tried to adjust the ratio from 60:40 to 40:60 to increase the number of samples in the validation set relatively. For binary and multiclass sample classification, a SVM algorithm implemented in Python 3.7.2 was used. Particularly, we applied a One-Versus-One approach to the classification algorithm. The algorithm’s accuracy reflects the percentage of correct predictions. In principle, the algorithm was trained on the training set to fix SVM parameters before the validation set was predicted.

## Results

### Platelet purity and algorithm details

Quantification of platelet purity of the used samples was not conducted initially when we carried out the study. To support the credibility of our isolation results, 10 newly collected blood samples were applied to platelet isolation following the protocol described above. The contamination of hemocytes was quantified using a hematology analyzer, yielding an average platelet purity of 93.7% (Table S1).

Per the ratio mentioned above, sizes of the training set and the validation set were fixed to be 103 and 69. Samples in each sample class were also extracted per this ratio according the total size of their classes to fill the two sets. For the binary algorithm, the training set contains 67 patient samples and 36 normal samples, and the validation set contains 42 patient samples and 27 normal samples. For the multiclass algorithm, the training set contains 20 TA, 26 PTC, 23 MPTC and 34 normal samples, and the validation set contains 12 TA, 15 PTC, 13 MPTC and 29 normal samples. Sample details were listed in Table 1.

**Table 1:** Baseline characteristics of subjects

	Healthy	TA	PTC	MPTC
Characteristic	(*n* = 63)	(*n* = 32)	(*n* = 41)	(*n* = 36)
Age, year				
Mean	50.4	58.9	47.5	43
Range	18–75	27–81	23–73	24–73
Sex, NO.(%)				
Female	34 (54%)	23 (72%)	33 (80%)	27 (75%)
Male	29 (46%)	9 (28%)	8 (20%)	9 (25%)
One or both sides affected				
One	0	13	30	12
Both	0	19	11	24
lymph node metastasis				
Negative	63	32	41	0
Positive	0	0	0	36
Level VI lymph node metastasis				
Negative	63	32	41	0
Positive	0	0	0	36
Lateral lymph node metastasis				
Negative	63	32	41	23
Positive	0	0	0	13
Distant metastasis				
Negative	63	32	41	36
Positive	0	0	0	0

*TA* thyroid adenomas, *PTC* papillary thyroid cancer, *MPTC* metastasized papillary thyroid cancer

### Gene expression profiles of platelets of patients are distinct from platelets of healthy individuals

Initially, 287 thyroid neoplasm patients were recruited. 119 patients with comorbidities and 58 patients with thyroid nodules were excluded before blood drawn, and 1 patient whose transcript profile contains too many zero was excluded before SVM training. Baseline characteristics of 172 subjects are described in Table 2 and a more detailed version is shown in Table S2. Platelets were collected and isolated from healthy individuals (*n* = 63) and patients with thyroid adenomas (*n* = 32), papillary thyroid cancer (*n* = 41) and metastasized papillary thyroid cancer (*n* = 36). Platelet RNA-seq yielded a mean read count of ~18.8 million reads per sample, and a total of 58302 RNAs were detected in the dataset. We identified 765 GDTLs (base mean > 100, |log2FC| > 1, FDR < 0.05) in platelets of healthy individuals (*n* = 63) and patients (*n* = 109), 442 with higher and 323 with lower expression (Table 3).

**Table 2:** Genes with differential transcript levels between healthy donor platelets and TEPs

Ensembl gene ID	Gene name	Base mean	Log2FoldChange	LfcSE	Stat	*P* value	*P*adj
ENSG00000167972	ABCA3	147.744335	−1.193301558	0.310807435	−3.839359762	0.000123356	0.001392927
ENSG00000146386	ABRACL	333.7143949	1.058477961	0.194479886	5.442608923	5.25E−08	1.67E−06
ENSG00000248242	AC004053.1	286.964615	1.073157894	0.180392513	5.949015725	2.70E−09	1.17E−07
ENSG00000278730	AC005332.9	270.9781692	1.294118644	0.211712906	6.112611016	9.80E−10	4.71E−08
ENSG00000260078	AC007342.3	105.1375405	2.093197408	0.311531581	6.719053666	1.83E−11	1.33E−09
ENSG00000198134	AC007537.1	271.6173226	2.281619991	0.154744785	14.74440632	3.34E−49	7.33E−45
ENSG00000282828	AC009971.1	365.524061	1.096814555	0.200711282	5.464638273	4.64E−08	1.50E−06
ENSG00000282863	AC012560.1	176.9862285	1.380186203	0.2158771	6.393388646	1.62E−10	9.44E−09
ENSG00000229056	AC020571.1	156.8912964	1.322281005	0.284973242	4.640018114	3.48E−06	6.53E−05
ENSG00000276900	AC023157.3	200.1065793	1.554961048	0.28086639	5.536301616	3.09E−08	1.05E−06
ENSG00000253683	AC027309.2	503.0465298	1.00317469	0.138384755	7.249170551	4.19E−13	4.46E−11
ENSG00000228651	AC074327.1	958.1155626	1.366733603	0.175066594	7.806935452	5.86E−15	9.18E−13
ENSG00000267279	AC090409.1	2450.181507	1.272765796	0.151453598	8.403668261	4.33E−17	1.05E−14
ENSG00000267316	AC090409.2	640.4657687	1.236821945	0.187893512	6.582568662	4.62E−11	3.05E−09
ENSG00000266356	AC090615.1	533.7245751	1.565537836	0.173839184	9.005667217	2.14E−19	7.05E−17
ENSG00000242611	AC093627.6	102.8967931	−1.019355916	0.335988942	−3.033897217	0.002414167	0.016232838
ENSG00000260244	AC104083.1	186.7275242	2.250433112	0.286323124	7.85976724	3.85E−15	6.25E−13
ENSG00000224550	AC114491.1	1103.377462	1.422509807	0.156502424	9.089378754	9.96E−20	3.47E−17
ENSG00000270055	AC127502.2	914.2748041	1.294074642	0.202096009	6.403266703	1.52E−10	8.87E−09
ENSG00000268027	AC243960.1	158.807324	1.34975385	0.285009962	4.735812879	2.18E−06	4.38E−05
ENSG00000274265	AC245297.3	235.8684255	1.051080891	0.136661311	7.691137171	1.46E−14	2.10E−12
ENSG00000072778	ACADVL	763.5822244	−1.038153695	0.252120613	−4.117686697	3.83E−05	0.000512965
ENSG00000213763	ACTBP2	775.2718294	−2.454491122	0.195058917	−12.58333205	2.61E−36	1.14E−32
ENSG00000196839	ADA	102.6507672	−1.254988764	0.39296447	−3.19364436	0.001404891	0.010373013
ENSG00000205336	ADGRG1	220.5487915	−1.106319443	0.401914822	−2.752621653	0.005912018	0.032963294
ENSG00000006831	ADIPOR2	189.1574602	1.163775143	0.263933052	4.409357345	1.04E−05	0.000168786
ENSG00000148926	ADM	148.9918432	1.33970977	0.42443462	3.156457337	0.001596982	0.011588428
ENSG00000101126	ADNP	663.6374206	2.732546294	0.304178002	8.983379051	2.63E−19	8.34E−17
ENSG00000139154	AEBP2	137.7554643	2.385952666	0.387242159	6.161396969	7.21E−10	3.66E−08
ENSG00000104964	AES	401.459888	−1.476859609	0.276360879	−5.343953223	9.09E−08	2.73E−06
ENSG00000153207	AHCTF1	374.6087716	1.395380759	0.177058094	7.8809205	3.25E−15	5.40E−13
ENSG00000169877	AHSP	146.5475537	2.460662952	0.414786412	5.932361529	2.99E−09	1.29E−07
ENSG00000197976	AKAP17A	107.697046	−1.46963743	0.403266187	−3.644335864	0.000268083	0.002643343
ENSG00000127914	AKAP9	577.7550486	1.587197006	0.192453222	8.247183345	1.62E−16	3.39E−14
ENSG00000117020	AKT3	105.6687378	1.015720444	0.311658516	3.259081306	0.001117736	0.008612382
ENSG00000284633	AL031590.1	145.3481615	1.016195682	0.288012364	3.528305751	0.000418229	0.003848652
ENSG00000260257	AL035071.1	105.1746625	1.831420904	0.410889297	4.457212489	8.30E−06	0.000138956
ENSG00000260912	AL158206.1	2262.742941	2.595114724	0.27388215	9.47529702	2.66E−21	1.19E−18
ENSG00000259834	AL365361.1	141.2560278	2.911450043	0.276029926	10.54758841	5.21E−26	4.57E−23
ENSG00000234810	AL603840.1	1095.597029	1.023702274	0.177094182	5.780552815	7.45E−09	3.01E−07
ENSG00000132965	ALOX5AP	1081.09253	1.321655277	0.276291063	4.783561446	1.72E−06	3.59E−05
ENSG00000129055	ANAPC13	237.1757126	1.157183739	0.227761384	5.080684514	3.76E−07	9.55E−06
ENSG00000154188	ANGPT1	766.2254775	1.00880793	0.195846311	5.151018304	2.59E−07	6.80E−06
ENSG00000189045	ANKDD1B	218.8923211	1.050998661	0.25244561	4.163267732	3.14E−05	0.000436528
ENSG00000156381	ANKRD9	432.1759845	−1.033952456	0.329667657	−3.136347877	0.001710662	0.01226319
ENSG00000143401	ANP32E	581.310852	2.513928153	0.318696419	7.888159396	3.07E−15	5.13E−13
ENSG00000122359	ANXA11	1415.225151	−1.149432797	0.202645607	−5.672132814	1.41E−08	5.31E−07
ENSG00000283633	AP000547.3	2246.545197	−1.122502149	0.122502206	−9.163117855	5.04E−20	1.79E−17
ENSG00000254786	AP001636.1	253.4004781	1.113737449	0.160594411	6.935094714	4.06E−12	3.45E−10
ENSG00000254614	AP003068.2	2470.376513	−1.182928739	0.155196426	−7.622139054	2.50E−14	3.48E−12
ENSG00000100280	AP1B1	606.4329023	−1.060492311	0.25396113	−4.175805601	2.97E−05	0.000416866
ENSG00000166747	AP1G1	270.7352712	1.015829553	0.221796689	4.580003225	4.65E−06	8.35E−05
ENSG00000196961	AP2A1	113.5934072	−1.64002848	0.368914541	−4.445551196	8.77E−06	0.000145972
ENSG00000242802	AP5Z1	108.9648119	−1.164844966	0.40346742	−2.887085567	0.003888285	0.023724595
ENSG00000128335	APOL2	217.3825445	1.540755916	0.310633656	4.960041795	7.05E−07	1.66E−05
ENSG00000221963	APOL6	140.4867515	1.123177512	0.324247277	3.463953565	0.000532298	0.004674718
ENSG00000143595	AQP10	517.6872055	−1.368736476	0.252535652	−5.419973231	5.96E−08	1.87E−06
ENSG00000103569	AQP9	384.0466257	1.742015624	0.352543814	4.941274125	7.76E−07	1.80E−05
ENSG00000021776	AQR	118.5240046	−1.094240298	0.356742023	−3.06731539	0.002159908	0.014847846
ENSG00000165527	ARF6	714.2496746	1.11296045	0.292576657	3.803996051	0.00014238	0.001569764
ENSG00000140750	ARHGAP17	194.53761	−1.262139206	0.322044896	−3.919140531	8.89E−05	0.001051661
ENSG00000146376	ARHGAP18	6922.149294	1.033544846	0.124801853	8.281486364	1.22E−16	2.62E−14
ENSG00000107863	ARHGAP21	536.6234917	1.128264204	0.186472975	6.050550778	1.44E−09	6.54E−08
ENSG00000159314	ARHGAP27	220.509626	−1.035054736	0.36250939	−2.855249449	0.004300304	0.025744298
ENSG00000134909	ARHGAP32	114.4259962	1.042184206	0.231797691	4.496094	6.92E−06	0.000118023
ENSG00000089820	ARHGAP4	396.4862759	−1.048102647	0.2982872	−3.513736586	0.000441851	0.004021864
ENSG00000180448	ARHGAP45	2893.235745	−1.308221023	0.245219129	−5.334906093	9.56E−08	2.85E−06
ENSG00000141522	ARHGDIA	213.1310664	−1.664317465	0.327042046	−5.089001512	3.60E−07	9.17E−06
ENSG00000076928	ARHGEF1	457.4885964	−1.153432015	0.333366759	−3.459949093	0.000540278	0.004734605
ENSG00000054267	ARID4B	374.641917	1.369063187	0.246027805	5.564668548	2.63E−08	9.17E−07
ENSG00000196843	ARID5A	337.6413652	−1.118653087	0.318932565	−3.507490959	0.000452354	0.00410073
ENSG00000150347	ARID5B	247.308519	1.103126901	0.326044793	3.383359973	0.000716047	0.00597041
ENSG00000130429	ARPC1B	230.2471289	−1.164119058	0.141189376	−8.245089635	1.65E−16	3.41E−14
ENSG00000241553	ARPC4	5512.938756	1.206587203	0.169207537	7.130812384	9.98E−13	9.86E−11
ENSG00000141480	ARRB2	613.1271772	−1.159715331	0.280321494	−4.137090291	3.52E−05	0.000478826
ENSG00000105643	ARRDC2	123.8856016	−1.167587121	0.360422508	−3.239495581	0.001197413	0.009114222
ENSG00000151693	ASAP2	325.9074825	−1.171306595	0.224535007	−5.216587886	1.82E−07	5.00E−06
ENSG00000198925	ATG9A	473.9360086	−1.184673823	0.247678006	−4.783120801	1.73E−06	3.60E−05
ENSG00000074370	ATP2A3	3314.980545	−1.530610551	0.187151233	−8.178468964	2.87E−16	5.68E−14
ENSG00000100554	ATP6V1D	695.1547879	1.008349107	0.154818403	6.513108839	7.36E−11	4.55E−09
ENSG00000123472	ATPAF1	234.2959111	−1.202396182	0.289057805	−4.159708413	3.19E−05	0.000442264
ENSG00000085224	ATRX	494.6608028	1.515672106	0.22004679	6.887953714	5.66E−12	4.63E−10
ENSG00000124788	ATXN1	112.8709901	1.438463803	0.326258741	4.408966324	1.04E−05	0.000168966
ENSG00000156735	BAG4	168.1303046	1.457913524	0.31667489	4.603817884	4.15E−06	7.54E−05
ENSG00000138376	BARD1	252.4751454	1.560642584	0.219727861	7.102615833	1.22E−12	1.19E−10
ENSG00000198604	BAZ1A	209.6754244	1.451131249	0.224813466	6.454823535	1.08E−10	6.49E−09
ENSG00000123636	BAZ2B	212.1858341	1.215930277	0.306997288	3.960719934	7.47E−05	0.000908692
ENSG00000114439	BBX	635.2260958	1.281293819	0.190316338	6.732442596	1.67E−11	1.24E−09
ENSG00000142867	BCL10	218.6358006	1.487582109	0.294128094	5.057599521	4.25E−07	1.07E−05
ENSG00000140379	BCL2A1	652.4756833	2.04855901	0.276880213	7.398719429	1.38E−13	1.66E−11
ENSG00000106635	BCL7B	393.7922008	−1.100379028	0.251883546	−4.368602264	1.25E−05	0.000199031
ENSG00000186716	BCR	315.7952402	1.110964431	0.178803269	6.213334017	5.19E−10	2.70E−08
ENSG00000023445	BIRC3	358.0950135	1.40657258	0.303490819	4.634646237	3.58E−06	6.66E−05
ENSG00000090013	BLVRB	636.5815679	1.244806773	0.240521032	5.175459131	2.27E−07	6.06E−06
ENSG00000038219	BOD1L1	146.7957058	1.823203983	0.330185326	5.521759574	3.36E−08	1.13E−06
ENSG00000171634	BPTF	342.5067721	1.210283409	0.229541296	5.272617297	1.34E−07	3.83E−06
ENSG00000113460	BRIX1	104.2246174	1.119986592	0.374005907	2.994569258	0.002748327	0.017931584
ENSG00000162819	BROX	2039.076685	1.67089885	0.235380715	7.098707509	1.26E−12	1.22E−10
ENSG00000064726	BTBD1	232.5646723	−1.70792708	0.278905283	−6.123681346	9.14E−10	4.45E−08
ENSG00000134717	BTF3L4	124.0449488	1.171530452	0.276118578	4.242852689	2.21E−05	0.000323939
ENSG00000171067	C11orf24	162.2211041	−1.214168111	0.290091528	−4.185465603	2.85E−05	0.000401325
ENSG00000235162	C12orf75	4093.060399	−1.324146142	0.127631049	−10.37479632	3.23E−25	2.53E−22
ENSG00000156411	C14orf2	1367.017358	1.110983647	0.142417112	7.800914041	6.15E−15	9.49E−13
ENSG00000185905	C16orf54	872.9524918	1.122240564	0.281094052	3.992402381	6.54E−05	0.000810443
ENSG00000119280	C1orf198	2354.804675	−1.044882608	0.172596826	−6.053892357	1.41E–09	6.42E−08
ENSG00000082213	C5orf22	109.0119095	−1.4621057	0.3608888	−4.051402263	5.09E−05	0.000654037
ENSG00000112308	C6orf62	11385.09564	2.708976737	0.259435007	10.44183193	1.60E−25	1.35E−22
ENSG00000133742	CA1	265.7929404	1.795055302	0.358481485	5.007386377	5.52E−07	1.34E−05
ENSG00000151893	CACUL1	618.9970501	1.093175467	0.264648251	4.130673305	3.62E−05	0.000490221
ENSG00000143933	CALM2	9984.484119	1.269216343	0.136470889	9.300271685	1.40E−20	5.49E−18
ENSG00000164047	CAMP	106.7789662	1.850629594	0.44375531	4.170382984	3.04E−05	0.000424465
ENSG00000108509	CAMTA2	148.3957162	−1.642107327	0.307292722	−5.343788534	9.10E−08	2.73E−06
ENSG00000014216	CAPN1	4327.495514	−1.105564373	0.207857232	−5.318864123	1.04E−07	3.07E−06
ENSG00000116489	CAPZA1	4091.417875	2.183614904	0.23157686	9.429331187	4.13E−21	1.77E−18
ENSG00000204397	CARD16	2295.713559	1.153498287	0.181277832	6.363151398	1.98E−10	1.13E−08
ENSG00000079691	CARMIL1	150.0064564	1.935304361	0.288345377	6.711757906	1.92E−11	1.40E−09
ENSG00000106144	CASP2	1057.831786	1.70724393	0.247335264	6.902549613	5.11E−12	4.24E−10
ENSG00000164305	CASP3	531.6863406	1.183011247	0.193667634	6.108461292	1.01E−09	4.81E−08
ENSG00000196954	CASP4	1422.940309	1.567585966	0.189105498	8.289478526	1.14E−16	2.49E−14
ENSG00000105879	CBLL1	272.7062747	−1.038772815	0.328230439	−3.164766862	0.001552073	0.011307442
ENSG00000160799	CCDC12	246.0327546	1.167430604	0.25657993	4.549968522	5.37E−06	9.44E−05
ENSG00000151838	CCDC175	138.7646967	3.032416702	0.26957477	11.24888913	2.35E−29	3.67E−26
ENSG00000122483	CCDC18	184.7280627	2.890832479	0.243000652	11.89639804	1.24E−32	3.08E−29
ENSG00000198624	CCDC69	298.7204061	−1.115008094	0.288446692	−3.865560345	0.000110834	0.001270511
ENSG00000149231	CCDC82	293.4912716	1.35963164	0.186147135	7.304069661	2.79E−13	3.06E−11
ENSG00000175602	CCDC85B	903.9924242	−1.111989796	0.338777128	−3.282363839	0.001029407	0.008053465
ENSG00000276070	CCL4L2	235.8379994	2.233657151	0.353220837	6.323684561	2.55E−10	1.43E−08
ENSG00000126353	CCR7	545.3391503	1.144478631	0.361855147	3.16280877	0.001562549	0.011372434
ENSG00000177697	CD151	1737.632158	−1.948204342	0.198290838	−9.82498418	8.79E−23	4.94E−20
ENSG00000116824	CD2	300.9721829	1.295584434	0.346076695	3.743633864	0.000181378	0.001925187
ENSG00000160654	CD3G	425.2286981	1.180168365	0.332075154	3.553919502	0.000379535	0.003552438
ENSG00000173762	CD7	103.9339593	−1.144764977	0.389799943	−2.936801298	0.003316165	0.020890602
ENSG00000151465	CDC123	196.8026183	1.061714028	0.287463202	3.693391089	0.000221283	0.00226014
ENSG00000070831	CDC42	7117.683974	1.81802314	0.240088009	7.572319631	3.67E−14	4.90E−12
ENSG00000158985	CDC42SE2	12344.8955	1.685660978	0.236361675	7.131701771	9.91E−13	9.84E−11
ENSG00000103502	CDIPT	325.2438595	−1.050049838	0.291208306	−3.605837528	0.000311148	0.002995241
ENSG00000102225	CDK16	356.9771492	−1.045183689	0.252193025	−4.144379836	3.41E−05	0.000466706
ENSG00000155111	CDK19	159.6436276	1.428227007	0.226254993	6.312466249	2.75E−10	1.52E−08
ENSG00000145996	CDKAL1	138.4920286	1.110943353	0.276904752	4.012005379	6.02E−05	0.000753131
ENSG00000091527	CDV3	271.8124416	1.813048995	0.234421214	7.734150682	1.04E−14	1.54E−12
ENSG00000170956	CEACAM3	116.2416158	1.097008908	0.409496791	2.678919426	0.007386016	0.039113247
ENSG00000105352	CEACAM4	115.0091782	1.096327728	0.410354064	2.671662898	0.007547643	0.03969119
ENSG00000135315	CEP162	266.5333126	1.01294131	0.221358678	4.576018065	4.74E−06	8.48E−05
ENSG00000182923	CEP63	140.7908205	1.424681687	0.305061813	4.670141024	3.01E−06	5.77E−05
ENSG00000124177	CHD6	116.937282	1.149940153	0.245963125	4.675254275	2.94E−06	5.66E−05
ENSG00000133048	CHI3L1	140.0476038	1.375870657	0.499462994	2.754699893	0.005874599	0.032829788
ENSG00000130724	CHMP2A	779.230596	1.013333907	0.165660384	6.116935625	9.54E−10	4.61E−08
ENSG00000187446	CHP1	1405.964711	1.255352417	0.230957594	5.435423865	5.47E−08	1.73E−06
ENSG00000120903	CHRNA2	167.5322889	−1.060685446	0.30801255	−3.443643596	0.000573932	0.004972639
ENSG00000124302	CHST8	225.8878062	−1.545551127	0.363015664	−4.25753288	2.07E−05	0.000305639
ENSG00000179583	CIITA	143.5895847	−1.474239773	0.389235449	−3.787527007	0.000152154	0.001659994
ENSG00000138433	CIR1	256.5173867	1.566378568	0.223434932	7.010446205	2.38E−12	2.18E−10
ENSG00000105205	CLC	116.6933762	1.556528267	0.336257158	4.628981808	3.67E−06	6.81E−05
ENSG00000110852	CLEC2B	202.1129023	1.125483229	0.27659869	4.069011428	4.72E−05	0.000614077
ENSG00000166523	CLEC4E	143.8630559	1.700251286	0.369116234	4.606276099	4.10E−06	7.47E−05
ENSG00000172243	CLEC7A	408.345313	1.179311852	0.300315324	3.92691201	8.60E−05	0.001023236
ENSG00000104853	CLPTM1	101.5765257	−1.677524205	0.402747806	−4.165197623	3.11E−05	0.000433677
ENSG00000162368	CMPK1	6337.106446	−1.044231988	0.11943044	−8.743432485	2.26E−18	6.61E−16
ENSG00000140932	CMTM2	381.959462	1.463676077	0.216453569	6.762078749	1.36E−11	1.04E−09
ENSG00000140931	CMTM3	279.7016359	−1.43668811	0.272898166	−5.264557592	1.41E−07	3.98E−06
ENSG00000143771	CNIH4	158.2432116	1.490313925	0.277899142	5.362787058	8.19E−08	2.50E−06
ENSG00000144580	CNOT9	291.0274522	1.211771691	0.288315695	4.202933493	2.63E−05	0.000375426
ENSG00000044459	CNTLN	118.2814154	1.952512659	0.269735601	7.238616826	4.53E−13	4.73E−11
ENSG00000183978	COA3	264.1133391	1.039825689	0.20757087	5.009497182	5.46E−07	1.32E−05
ENSG00000145781	COMMD10	149.7552133	1.210806766	0.31810381	3.806325879	0.000141047	0.00155584
ENSG00000103187	COTL1	7983.640125	−1.180352021	0.171044471	−6.900848746	5.17E−12	4.26E−10
ENSG00000226976	COX6A1P2	497.4712853	1.167808426	0.172853126	6.756073515	1.42E−11	1.08E−09
ENSG00000103381	CPPED1	771.6448852	1.053225843	0.244244037	4.31218652	1.62E−05	0.000247159
ENSG00000095321	CRAT	1897.330471	−1.485504198	0.229467966	−6.473688799	9.56E−11	5.78E−09
ENSG00000146592	CREB5	142.6416224	1.076348317	0.352961858	3.04947487	0.002292418	0.015553972
ENSG00000143162	CREG1	1093.078971	−1.11572065	0.158429896	−7.042361799	1.89E−12	1.78E−10
ENSG00000103196	CRISPLD2	126.8032804	1.255576214	0.410373838	3.059591274	0.002216392	0.015145924
ENSG00000088766	CRLS1	303.9292194	−1.36356579	0.248671532	−5.483401255	4.17E−08	1.36E−06
ENSG00000062485	CS	243.2391339	−1.12952203	0.313828725	−3.599167128	0.000319238	0.003061027
ENSG00000169826	CSGALNACT2	125.1673522	−1.133985947	0.331791103	−3.417770814	0.000631362	0.005364257
ENSG00000141551	CSNK1D	185.4656384	−1.528694545	0.320826481	−4.764863984	1.89E−06	3.88E−05
ENSG00000151292	CSNK1G3	166.7871781	1.078606104	0.275773655	3.91120067	9.18E−05	0.001081012
ENSG00000070770	CSNK2A2	221.6677401	1.263176229	0.316099068	3.996140319	6.44E−05	0.00079902
ENSG00000178585	CTNNBIP1	134.1337189	−1.684890821	0.246279309	−6.84138195	7.84E−12	6.28E−10
ENSG00000085733	CTTN	24371.26154	1.274072698	0.191047469	6.668880282	2.58E−11	1.79E−09
ENSG00000163510	CWC22	114.5990004	1.475421636	0.33624907	4.387883168	1.14E−05	0.000184016
ENSG00000163739	CXCL1	119.7436881	1.437836338	0.444840442	3.232251838	0.001228188	0.009300044
ENSG00000163464	CXCR1	509.1232218	1.07287118	0.390865733	2.744858628	0.006053697	0.033599474
ENSG00000180871	CXCR2	1527.907552	1.962767851	0.304201945	6.452187059	1.10E−10	6.57E−09
ENSG00000173198	CYSLTR1	103.4504222	1.293326791	0.374922507	3.44958429	0.00056145	0.00487799
ENSG00000070190	DAPP1	18751.68305	1.016116153	0.118334802	8.586790486	8.94E−18	2.39E−15
ENSG00000198924	DCLRE1A	1810.995245	1.115386065	0.178959923	6.232602499	4.59E−10	2.44E−08
ENSG00000204843	DCTN1	148.5563947	−1.365900405	0.362757493	−3.765326507	0.000166332	0.001791496
ENSG00000043093	DCUN1D1	660.3994625	1.113485021	0.217929625	5.109378872	3.23E−07	8.36E−06
ENSG00000223972	DDX11L1	179.4122136	1.077603253	0.243001638	4.434551403	9.23E−06	0.000152354
ENSG00000104325	DECR1	282.5916113	1.05424058	0.225809323	4.668720338	3.03E−06	5.80E−05
ENSG00000239839	DEFA3	179.2155954	1.121946875	0.378701751	2.962613382	0.003050394	0.01953054
ENSG00000100150	DEPDC5	119.7234025	1.020496443	0.198321347	5.145671189	2.67E−07	6.99E−06
ENSG00000149091	DGKZ	196.061758	−1.201928097	0.263019852	−4.569723875	4.88E−06	8.71E−05
ENSG00000132153	DHX30	143.8416667	−1.107385314	0.304355706	−3.638457542	0.000274276	0.002693504
ENSG00000086189	DIMT1	1205.29441	1.052750092	0.157586763	6.680447475	2.38E−11	1.68E−09
ENSG00000080845	DLGAP4	111.0937391	−1.016515571	0.280873139	−3.619127038	0.000295599	0.002869491
ENSG00000174844	DNAH12	205.8110097	1.093313769	0.250481373	4.364850584	1.27E−05	0.00020229
ENSG00000102580	DNAJC3	617.1671568	2.131605303	0.305920122	6.967849279	3.22E−12	2.87E−10
ENSG00000134516	DOCK2	314.126723	−1.115916986	0.297971962	−3.74504024	0.000180365	0.001916289
ENSG00000147459	DOCK5	559.4103809	1.98257673	0.287123841	6.904953366	5.02E−12	4.20E−10
ENSG00000163840	DTX3L	214.7231261	1.334257586	0.337499725	3.953359026	7.71E−05	0.00093158
ENSG00000276023	DUSP14	129.2662912	−1.361866252	0.322054515	−4.228682374	2.35E−05	0.000340881
ENSG00000158050	DUSP2	436.7205155	−1.067194918	0.411049209	−2.596270459	0.009424185	0.046788081
ENSG00000157540	DYRK1A	106.071614	1.768395616	0.310225811	5.70034973	1.20E−08	4.59E−07
ENSG00000101412	E2F1	331.955714	−1.105975942	0.314946178	−3.511634744	0.00044536	0.004044013
ENSG00000144597	EAF1	349.7036266	1.758958213	0.286284678	6.144087847	8.04E−10	3.99E−08
ENSG00000145088	EAF2	270.0246978	1.714764657	0.226931103	7.55632275	4.15E−14	5.35E−12
ENSG00000178852	EFCAB13	230.6164389	2.439413703	0.25511484	9.562021948	1.15E−21	5.76E−19
ENSG00000090776	EFNB1	224.5218347	−1.224054755	0.292239833	−4.188528111	2.81E−05	0.000396714
ENSG00000181090	EHMT1	102.5689753	−1.143417583	0.315628215	−3.622672267	0.000291575	0.002837972
ENSG00000086232	EIF2AK1	5756.493461	−1.095169313	0.126194127	−8.678449142	4.01E−18	1.11E−15
ENSG00000130741	EIF2S3	979.4717907	2.00462787	0.288214145	6.955341742	3.52E−12	3.09E−10
ENSG00000063046	EIF4B	463.5127351	1.346254249	0.279886551	4.809999774	1.51E−06	3.21E−05
ENSG00000066044	ELAVL1	291.1125708	−1.500808941	0.244342411	−6.142236776	8.14E−10	4.03E−08
ENSG00000156030	ELMSAN1	437.6228871	1.977616204	0.357838376	5.526562656	3.27E−08	1.10E−06
ENSG00000164181	ELOVL7	2817.931307	−1.113997703	0.173120357	−6.434816345	1.24E−10	7.29E−09
ENSG00000128463	EMC4	369.3282879	1.11241857	0.241180756	4.612385292	3.98E−06	7.29E−05
ENSG00000102119	EMD	515.6826346	−1.926184102	0.20586822	−9.356393645	8.25E−21	3.48E−18
ENSG00000187266	EPOR	536.0635452	−1.031820773	0.235968016	−4.372714526	1.23E−05	0.000196132
ENSG00000100632	ERH	122.6904211	1.018390329	0.247557271	4.113756479	3.89E−05	0.000520283
ENSG00000197930	ERO1A	122.6362877	1.482506453	0.406781446	3.644479038	0.000267934	0.002643061
ENSG00000139163	ETNK1	399.3056231	1.536160948	0.301369637	5.097265146	3.45E−07	8.87E−06
ENSG00000185862	EVI2B	550.63855	1.815176033	0.26989607	6.725463003	1.75E-11	1.29E−09
ENSG00000120699	EXOSC8	159.5726713	1.358142973	0.343950406	3.948659308	7.86E−05	0.000947136
ENSG00000134824	FADS2	102.7609647	−1.448435121	0.278129685	−5.207768896	1.91E−07	5.19E−06
ENSG00000197798	FAM118B	160.038343	1.065094429	0.278908147	3.818799985	0.000134102	0.001490488
ENSG00000048828	FAM120A	210.2431013	1.656700188	0.251834661	6.578523304	4.75E−11	3.12E−09
ENSG00000112584	FAM120B	183.5329268	−1.014108371	0.33137616	−3.060293689	0.0022112	0.015119867
ENSG00000160767	FAM189B	171.1357322	−1.155310749	0.262771324	−4.396639377	1.10E−05	0.000177374
ENSG00000144649	FAM198A	721.5200567	1.055866019	0.172111217	6.134789111	8.53E−10	4.21E−08
ENSG00000157870	FAM213B	122.363102	−1.105518393	0.306003696	−3.61276157	0.000302953	0.002931801
ENSG00000108306	FBXL20	100.080468	1.120876901	0.36876981	3.039502882	0.002369689	0.015985471
ENSG00000072803	FBXW11	200.6104376	−1.314032315	0.250680263	−5.241865862	1.59E−07	4.45E−06
ENSG00000159069	FBXW5	1644.243652	−1.019681482	0.237068435	−4.301211506	1.70E−05	0.000257075
ENSG00000162747	FCGR3B	2049.710163	1.8696195	0.31667113	5.903978359	3.55E−09	1.52E−07
ENSG00000171055	FEZ2	123.1786647	2.182208862	0.229383633	9.513359039	1.85E−21	8.61E−19
ENSG00000126262	FFAR2	213.1753805	1.176719346	0.367921931	3.198285411	0.001382474	0.010245444
ENSG00000127951	FGL2	646.901379	1.64346734	0.298485237	5.506025541	3.67E−08	1.22E−06
ENSG00000100442	FKBP3	115.3609315	1.340417789	0.220451457	6.080330832	1.20E−09	5.61E−08
ENSG00000105701	FKBP8	3573.219851	−2.030414636	0.319756657	−6.349874479	2.15E−10	1.22E−08
ENSG00000217128	FNIP1	171.6905115	1.979816703	0.310096236	6.38452349	1.72E−10	9.97E−09
ENSG00000065970	FOXJ2	136.0990418	−1.05662968	0.303390366	−3.482739726	0.000496311	0.004412524
ENSG00000171049	FPR2	267.1555377	1.978716602	0.388802066	5.089264632	3.59E−07	9.17E−06
ENSG00000126391	FRMD8	263.6497225	−1.553862688	0.276147354	−5.626933106	1.83E−08	6.67E−07
ENSG00000082074	FYB1	9060.233462	1.493254153	0.132716	11.25150062	2.28E−29	3.67E−26
ENSG00000123689	G0S2	3112.699436	1.460514566	0.301499904	4.844162626	1.27E−06	2.76E−05
ENSG00000171298	GAA	185.5687469	−1.173980655	0.403900225	−2.906610549	0.003653678	0.022543354
ENSG00000178950	GAK	174.3783024	−1.094589567	0.313070274	−3.496306284	0.000471747	0.004236256
ENSG00000143641	GALNT2	192.5753721	−1.033826216	0.251876211	−4.104501223	4.05E−05	0.000538834
ENSG00000175857	GAPT	161.8375122	1.125150038	0.303790895	3.70369901	0.000212478	0.002188557
ENSG00000185340	GAS2L1	2596.063539	−1.378630495	0.255837001	−5.388706435	7.10E−08	2.20E−06
ENSG00000117228	GBP1	332.9360188	1.263269901	0.300090984	4.209622971	2.56E−05	0.000366386
ENSG00000162645	GBP2	433.6800562	1.388182521	0.28632868	4.84821333	1.25E−06	2.72E−05
ENSG00000115271	GCA	605.7988793	1.027161688	0.248913697	4.126577597	3.68E−05	0.000497186
ENSG00000103365	GGA2	214.1192978	−1.133818214	0.324228977	−3.496967564	0.000470579	0.0042275
ENSG00000133574	GIMAP4	681.6526879	1.546112187	0.213212755	7.251499489	4.12E−13	4.41E−11
ENSG00000179144	GIMAP7	563.6636372	1.374704938	0.24839311	5.534392383	3.12E−08	1.06E−06
ENSG00000139436	GIT2	171.9789806	−1.011263775	0.26260221	−3.850933982	0.000117668	0.001336967
ENSG00000148672	GLUD1	173.7501689	−1.203086058	0.299178578	−4.021297469	5.79E−05	0.000729435
ENSG00000089639	GMIP	329.3233234	−1.222918232	0.275424739	−4.440117603	8.99E−06	0.000148802
ENSG00000060558	GNA15	406.4378805	−1.316165629	0.232715132	−5.655694227	1.55E−08	5.79E−07
ENSG00000156052	GNAQ	1225.259276	1.291001511	0.160637299	8.036748119	9.23E−16	1.69E−13
ENSG00000172354	GNB2	370.2844959	−1.405087512	0.285406538	−4.923109062	8.52E−07	1.95E−05
ENSG00000173905	GOLIM4	111.3621926	1.099746645	0.309417996	3.554242678	0.000379069	0.003552397
ENSG00000113384	GOLPH3	371.1546681	−1.854599988	0.229681802	−8.074649231	6.77E−16	1.26E−13
ENSG00000062194	GPBP1	530.8753953	1.030661581	0.203822061	5.056673326	4.27E−07	1.07E−05
ENSG00000198522	GPN1	119.9456645	1.037309172	0.332309961	3.12151092	0.001799256	0.012806193
ENSG00000111231	GPN3	118.8462346	1.09399601	0.201786821	5.421543418	5.91E−08	1.85E−06
ENSG00000173264	GPR137	299.293297	−1.848105148	0.31845417	−5.803362999	6.50E−09	2.65E−07
ENSG00000077585	GPR137B	192.2548746	−1.332478239	0.234937197	−5.671635889	1.41E−08	5.32E−07
ENSG00000140030	GPR65	148.897085	1.751799271	0.331513733	5.284243444	1.26E−07	3.62E−06
ENSG00000178719	GRINA	182.1151166	−1.189309191	0.30195291	−3.938724063	8.19E−05	0.000981069
ENSG00000198055	GRK6	826.7593475	−1.46152393	0.273240301	−5.348859325	8.85E−08	2.68E−06
ENSG00000104687	GSR	179.0999946	−1.338753693	0.277930635	−4.816862642	1.46E−06	3.11E−05
ENSG00000243955	GSTA1	230.4545639	1.460570825	0.333461843	4.380023841	1.19E−05	0.0001905
ENSG00000223622	GSTA6P	378.8060139	1.019956858	0.181114892	5.631546062	1.79E−08	6.51E−07
ENSG00000077235	GTF3C1	150.8368736	−1.044648416	0.380755648	−2.743618962	0.006076603	0.033692495
ENSG00000145649	GZMA	596.7086922	1.104068168	0.253746089	4.351074624	1.35E−05	0.000213736
ENSG00000132475	H3F3B	18695.10909	1.452958209	0.166954428	8.70272339	3.24E−18	9.23E−16
ENSG00000188921	HACD4	4716.887428	−1.065894028	0.136928508	−7.784310556	7.01E−15	1.07E−12
ENSG00000223609	HBD	610.1186404	1.459038917	0.295502295	4.937487594	7.91E−07	1.83E−05
ENSG00000206177	HBM	104.9212301	1.591992749	0.364253146	4.370566918	1.24E−05	0.000197783
ENSG00000182782	HCAR2	103.8665414	1.610033462	0.418251452	3.849439028	0.000118389	0.001344456
ENSG00000172534	HCFC1	175.2001099	−1.044279465	0.310714848	−3.360893341	0.000776908	0.006366525
ENSG00000069998	HDHD5	107.7371532	−1.243015319	0.470483585	−2.641995083	0.008241925	0.042297491
ENSG00000112406	HECA	173.9928832	1.650888283	0.301330292	5.478666855	4.29E−08	1.39E−06
ENSG00000136929	HEMGN	2317.27813	1.557947085	0.202248678	7.703126155	1.33E−14	1.93E−12
ENSG00000185359	HGS	226.5470731	−1.025630226	0.36544197	−2.806547442	0.005007554	0.029042753
ENSG00000149196	HIKESHI	122.0912769	1.013494476	0.262944622	3.854402757	0.000116012	0.001320916
ENSG00000273802	HIST1H2BG	1596.151115	1.221499648	0.168816966	7.235645054	4.63E−13	4.82E−11
ENSG00000197061	HIST1H4C	632.9875965	1.476463375	0.221348423	6.670313502	2.55E−11	1.78E−09
ENSG00000158406	HIST1H4H	5571.006376	1.332400867	0.245273824	5.432299472	5.56E−08	1.76E−06
ENSG00000276180	HIST1H4I	1631.424459	1.160008386	0.118729822	9.770151821	1.51E−22	8.29E−20
ENSG00000137309	HMGA1	113.4248449	−1.294077348	0.302785808	−4.273903572	1.92E−05	0.000286539
ENSG00000132967	HMGB1P5	6678.223937	1.088483577	0.10026763	10.8557824	1.87E−27	2.05E−24
ENSG00000253954	HMGN1P38	250.3510195	1.053287468	0.132869637	7.927224698	2.24E−15	3.96E−13
ENSG00000198830	HMGN2	1262.557369	1.023632022	0.194400723	5.265577229	1.40E−07	3.96E−06
ENSG00000230330	HMGN2P3	170.1405117	1.3748419	0.180064441	7.635277068	2.25E−14	3.17E−12
ENSG00000169045	HNRNPH1	1605.450207	1.087027207	0.251822519	4.316640186	1.58E−05	0.000244306
ENSG00000105323	HNRNPUL1	400.2598234	−1.219886693	0.198883943	−6.133661035	8.59E−10	4.23E−08
ENSG00000115756	HPCAL1	1154.862381	−1.011147936	0.229637662	−4.403232143	1.07E−05	0.0001726
ENSG00000185122	HSF1	173.3343563	−1.083222678	0.390538448	−2.773664627	0.005542879	0.031456984
ENSG00000160888	IER2	564.9589337	−1.265273133	0.3128771	−4.043994061	5.25E−05	0.000672305
ENSG00000163565	IFI16	458.5644884	1.264082902	0.218740612	5.7789127	7.52E−09	3.03E−07
ENSG00000185745	IFIT1	356.9862216	2.166537985	0.385050077	5.626639528	1.84E−08	6.67E−07
ENSG00000119922	IFIT2	876.6133202	1.536304083	0.296719431	5.177632205	2.25E−07	5.99E−06
ENSG00000119917	IFIT3	756.9337649	1.776172262	0.321559113	5.52362596	3.32E−08	1.12E−06
ENSG00000140443	IGF1R	117.3511234	1.474303315	0.274888855	5.363270607	8.17E−08	2.50E−06
ENSG00000115457	IGFBP2	425.0258627	−1.04427265	0.40136945	−2.601774126	0.009274291	0.04623231
ENSG00000211890	IGHA2	375.0040182	−1.240761331	0.352507842	−3.519811995	0.000431853	0.003955764
ENSG00000211896	IGHG1	818.968034	−1.181779236	0.316666332	−3.731938373	0.000190012	0.001999865
ENSG00000211651	IGLV1–44	123.1921406	1.286690651	0.350901342	3.66681599	0.000245589	0.002461394
ENSG00000140749	IGSF6	849.7293091	2.734892612	0.307898728	8.882442075	6.54E−19	2.02E−16
ENSG00000125538	IL1B	282.1358851	1.022614951	0.381264392	2.682167472	0.007314683	0.038837504
ENSG00000115590	IL1R2	150.177587	1.317023219	0.426441688	3.088401665	0.002012363	0.014062812
ENSG00000136689	IL1RN	263.0692957	1.206687631	0.369819293	3.262911518	0.001102739	0.008517778
ENSG00000100385	IL2RB	314.9632423	−1.525953063	0.402586578	−3.790372425	0.000150422	0.001643877
ENSG00000168685	IL7R	539.429256	1.185665977	0.329857845	3.594475608	0.000325046	0.003107204
ENSG00000106348	IMPDH1	290.5169148	−1.291488416	0.363466117	−3.553256701	0.000380493	0.003558137
ENSG00000259330	INAFM2	848.8102964	1.022094952	0.143750862	7.11018313	1.16E−12	1.13E−10
ENSG00000153487	ING1	120.5663775	1.280513841	0.254169842	5.038024305	4.70E−07	1.16E−05
ENSG00000168918	INPP5D	461.4918639	−1.185600555	0.317749828	−3.73123901	0.00019054	0.002002089
ENSG00000185507	IRF7	372.5129129	−1.349454151	0.365840988	−3.688635764	0.00022546	0.002293185
ENSG00000140968	IRF8	651.453882	−1.417304299	0.302254201	−4.689113644	2.74E−06	5.32E−05
ENSG00000172183	ISG20	769.9872187	1.027200598	0.226646532	4.532169938	5.84E−06	0.000101606
ENSG00000164171	ITGA2	130.4052439	1.504286346	0.275958263	5.451137179	5.00E−08	1.61E−06
ENSG00000259207	ITGB3	5845.184819	−1.452235259	0.158105489	−9.185229878	4.11E−20	1.50E−17
ENSG00000139626	ITGB7	112.9930354	−1.614720984	0.467544206	−3.453622056	0.000553112	0.004821498
ENSG00000135916	ITM2C	188.5520057	−2.05900138	0.475505644	−4.33013026	1.49E−05	0.000231601
ENSG00000100605	ITPK1	137.5400501	−1.098539939	0.320897119	−3.423339985	0.000618567	0.005280088
ENSG00000161677	JOSD2	112.1980605	−1.111592358	0.307010944	−3.620692944	0.000293815	0.002854705
ENSG00000177606	JUN	161.1988054	−1.755233628	0.394119796	−4.45355358	8.45E−06	0.000140954
ENSG00000171223	JUNB	1576.827226	−1.482918162	0.336302161	−4.409481511	1.04E−05	0.000168786
ENSG00000114166	KAT2B	129.8269942	1.203087865	0.298372559	4.032166593	5.53E−05	0.000701544
ENSG00000177272	KCNA3	402.7449592	−2.240614554	0.309322911	−7.24361008	4.37E−13	4.61E−11
ENSG00000069424	KCNAB2	253.4849401	−1.10581988	0.293222376	−3.771267029	0.000162421	0.001754544
ENSG00000171385	KCND3	345.1439369	1.80756237	0.260412632	6.941147031	3.89E−12	3.33E−10
ENSG00000157551	KCNJ15	133.9693962	1.707433598	0.444785627	3.838778717	0.000123648	0.001395508
ENSG00000123700	KCNJ2	152.1989559	3.693172804	0.527781098	6.997546551	2.60E−12	2.38E−10
ENSG00000120733	KDM3B	111.6918702	1.068863127	0.300754301	3.553941292	0.000379504	0.003552438
ENSG00000073614	KDM5A	398.9835472	1.209416417	0.236189225	5.120540184	3.05E−07	7.93E−06
ENSG00000183354	KIAA2026	103.2660779	1.329528301	0.305155145	4.356892959	1.32E−05	0.000209025
ENSG00000138182	KIF20B	107.668023	2.22165082	0.297310154	7.472502332	7.87E−14	9.64E−12
ENSG00000140859	KIFC3	713.10414	−1.006873083	0.223566903	−4.503676836	6.68E−06	0.000114332
ENSG00000155090	KLF10	369.781327	−1.048622006	0.330587991	−3.171990615	0.001513979	0.01106618
ENSG00000185896	LAMP1	208.2601598	−1.565937751	0.229224967	−6.831444986	8.41E−12	6.65E−10
ENSG00000188186	LAMTOR4	1425.810779	1.179889602	0.188647085	6.254480978	3.99E−10	2.14E−08
ENSG00000155506	LARP1	183.4331464	1.020452055	0.226604961	4.503220272	6.69E−06	0.000114488
ENSG00000196233	LCOR	1076.074824	1.319371393	0.197980326	6.664154076	2.66E−11	1.84E−09
ENSG00000157978	LDLRAP1	3748.221107	−1.343537929	0.189882241	−7.075637639	1.49E−12	1.42E−10
ENSG00000167615	LENG8	116.5777943	−1.964488656	0.362565637	−5.41829797	6.02E−08	1.88E−06
ENSG00000239961	LILRA4	110.4333076	−3.518698894	0.448780009	−7.840587429	4.48E−15	7.23E−13
ENSG00000251301	LINC02384	599.0593991	1.160457009	0.157821428	7.352974949	1.94E−13	2.25E−11
ENSG00000163898	LIPH	409.3840394	1.454656935	0.21610689	6.73119185	1.68E−11	1.25E−09
ENSG00000105983	LMBR1	102.2829949	−2.070338022	0.334038257	−6.197906917	5.72E−10	2.93E−08
ENSG00000139679	LPAR6	122.1289154	1.550485316	0.37907134	4.090220365	4.31E−05	0.000568628
ENSG00000171236	LRG1	138.4428419	1.369966715	0.423922443	3.231644698	0.0012308	0.00931018
ENSG00000197324	LRP10	1433.35877	−1.492053258	0.215858496	−6.912182227	4.77E−12	4.01E−10
ENSG00000129295	LRRC6	202.0750933	1.08930807	0.230418177	4.727526646	2.27E−06	4.52E−05
ENSG00000197147	LRRC8B	771.2554722	−1.106346371	0.200763176	−5.510703679	3.57E−08	1.19E−06
ENSG00000171492	LRRC8D	1135.940435	−1.267783043	0.182533125	−6.945495728	3.77E−12	3.26E−10
ENSG00000216863	LY86-AS1	356.2120761	1.074260661	0.194695611	5.517641911	3.44E−08	1.15E−06
ENSG00000154589	LY96	138.4398204	2.252987875	0.325070959	6.930757164	4.19E−12	3.54E−10
ENSG00000124688	MAD2L1BP	387.3674943	1.126169427	0.211610729	5.321891911	1.03E−07	3.03E−06
ENSG00000179632	MAF1	1444.891999	−1.228754652	0.243129091	−5.053918669	4.33E−07	1.08E−05
ENSG00000111196	MAGOHB	1415.157553	1.805859649	0.200733919	8.996285499	2.33E−19	7.53E−17
ENSG00000196782	MAML3	557.9559158	1.939045468	0.229617547	8.444674616	3.05E−17	7.60E−15
ENSG00000198162	MAN1A2	765.0560338	2.152129166	0.197240943	10.91116849	1.02E−27	1.18E−24
ENSG00000104774	MAN2B1	176.9351812	−1.346320394	0.376971731	−3.571409422	0.000355065	0.003354687
ENSG00000140400	MAN2C1	144.7869222	−1.707384705	0.387963675	−4.400888062	1.08E−05	0.000174139
ENSG00000131711	MAP1B	972.5256969	2.197085059	0.282969147	7.764397936	8.20E−15	1.22E−12
ENSG00000169032	MAP2K1	190.4405592	−1.085286542	0.261822311	−4.145126286	3.40E−05	0.000465479
ENSG00000047849	MAP4	125.6305347	−1.076199754	0.354479862	−3.035996879	0.002397418	0.016150556
ENSG00000114738	MAPKAPK3	402.4933501	−1.204360231	0.279351024	−4.311279098	1.62E–05	0.000247856
ENSG00000277443	MARCKS	137.2779777	1.48868534	0.294985137	5.046645247	4.50E−07	1.12E−05
ENSG00000007264	MATK	138.6675635	−1.652656366	0.417429719	−3.95912483	7.52E−05	0.000913329
ENSG00000129071	MBD4	355.5943416	1.053038844	0.189255018	5.564126423	2.63E−08	9.19E−07
ENSG00000144893	MED12L	3871.533119	1.005423911	0.146245554	6.874902403	6.20E−12	5.06E−10
ENSG00000099917	MED15	200.6149485	−1.058392133	0.303106117	−3.491820429	0.000479741	0.004293973
ENSG00000116604	MEF2D	169.9841397	−1.451573384	0.290573534	−4.995545753	5.87E−07	1.41E−05
ENSG00000205639	MFSD2B	1290.299962	−1.489976827	0.214456692	−6.947681678	3.71E−12	3.23E−10
ENSG00000152127	MGAT5	150.6122174	1.135844013	0.252111279	4.505328029	6.63E−06	0.000113535
ENSG00000102858	MGRN1	116.3710076	−1.139105491	0.305991492	−3.72267047	0.000197127	0.002060364
ENSG00000135596	MICAL1	141.882478	−1.990604931	0.379033022	−5.251798174	1.51E−07	4.25E−06
ENSG00000198160	MIER1	888.6229566	1.470484097	0.21269019	6.913737279	4.72E−12	3.98E−10
ENSG00000141503	MINK1	650.9301672	−1.525043284	0.271719331	−5.612568228	1.99E−08	7.20E−07
ENSG00000196588	MKL1	175.0511698	−1.110157487	0.297356547	−3.733422038	0.000188896	0.001989574
ENSG00000108960	MMD	16025.26162	−1.165927729	0.133795131	−8.714276207	2.93E−18	8.44E−16
ENSG00000126005	MMP24OS	237.9819055	−1.221704291	0.268540805	−4.549417705	5.38E−06	9.46E−05
ENSG00000163563	MNDA	3424.09243	2.692348694	0.244908709	10.99327461	4.12E−28	5.31E−25
ENSG00000182208	MOB2	693.8489652	−1.536103089	0.281940634	−5.448321041	5.08E−08	1.63E−06
ENSG00000196199	MPHOSPH8	286.4944627	1.278851359	0.253754788	5.039713216	4.66E−07	1.15E−05
ENSG00000128309	MPST	954.5682537	−1.12846372	0.235685004	−4.788016636	1.68E−06	3.52E−05
ENSG00000160588	MPZL3	196.2307938	1.169612788	0.303265039	3.856734669	0.000114912	0.001311082
ENSG00000179010	MRFAP1	788.1553585	1.001502158	0.148551299	6.741793325	1.56E−11	1.17E−09
ENSG00000178988	MRFAP1L1	157.2019169	1.011210729	0.340730453	2.967773261	0.002999655	0.019290158
ENSG00000188895	MSL1	148.3657363	1.039777946	0.295760143	3.515612131	0.000438741	0.004005479
ENSG00000248527	MTATP6P1	29018.57477	−1.08244429	0.189043462	−5.725901761	1.03E−08	4.02E−07
ENSG00000228253	MT-ATP8	3585.840493	−1.501847304	0.205413398	−7.311340539	2.64E−13	2.94E−11
ENSG00000137409	MTCH1	374.4988679	−1.31549824	0.305576542	−4.30497129	1.67E−05	0.000253096
ENSG00000198804	MT-CO1	148789.0531	−1.427469776	0.167258107	−8.534532677	1.41E−17	3.67E−15
ENSG00000237973	MTCO1P12	343.4435945	−2.522320964	0.199528098	−12.64143243	1.25E−36	6.84E−33
ENSG00000198938	MT-CO3	131137.1828	−1.032678521	0.177546141	−5.816395203	6.01E−09	2.49E−07
ENSG00000171100	MTM1	163.1182754	−1.187187433	0.323653563	−3.668080836	0.000244378	0.002450721
ENSG00000198888	MT-ND1	293611.6521	−1.221420515	0.18029823	−6.774445388	1.25E−11	9.64E−10
ENSG00000225630	MTND2P28	1529.929638	−1.954933299	0.202745704	−9.642292095	5.30E−22	2.70E−19
ENSG00000212907	MT-ND4L	22991.15887	−1.277249334	0.192885515	−6.62180016	3.55E−11	2.40E−09
ENSG00000210194	MT-TE	307.7871562	−1.632539745	0.242555063	−6.730594376	1.69E−11	1.25E−09
ENSG00000210049	MT-TF	103.0092337	−3.195633506	0.263293787	−12.13713984	6.71E−34	2.10E−30
ENSG00000209082	MT-TL1	220.3800993	−1.457336669	0.246438586	−5.913589643	3.35E−09	1.44E−07
ENSG00000210196	MT-TP	261.2071557	−1.233750041	0.183321262	−6.729988816	1.70E−11	1.25E−09
ENSG00000210195	MT-TT	122.6249109	−2.382516604	0.246817274	−9.652957276	4.78E−22	2.49E−19
ENSG00000210077	MT-TV	186.059006	−1.340670084	0.257604426	−5.204375199	1.95E−07	5.28E−06
ENSG00000059728	MXD1	875.6489733	1.630728695	0.234334087	6.958990536	3.43E−12	3.02E−10
ENSG00000179820	MYADM	209.6460162	−1.57683513	0.302692353	−5.209365593	1.89E−07	5.16E−06
ENSG00000136286	MYO1G	441.6352888	−1.091169497	0.320691432	−3.402552694	0.000667595	0.005643672
ENSG00000164134	NAA15	123.3193458	1.165563622	0.360993471	3.228766492	0.001243254	0.009389737
ENSG00000173559	NABP1	271.6571319	1.720596978	0.317473071	5.419662758	5.97E−08	1.87E−06
ENSG00000152620	NADK2	130.5396132	1.175795987	0.292989341	4.013101579	5.99E−05	0.000750497
ENSG00000105835	NAMPT	1727.500245	1.698957433	0.279469645	6.079219921	1.21E−09	5.63E−08
ENSG00000229644	NAMPTP1	478.3460557	1.73066928	0.27779032	6.230128104	4.66E−10	2.46E−08
ENSG00000132780	NASP	147.5036952	1.003628159	0.279744479	3.58766029	0.000333659	0.003175694
ENSG00000116701	NCF2	789.5355036	1.038833516	0.239203882	4.342878998	1.41E−05	0.000220714
ENSG00000213672	NCKIPSD	695.7097236	−1.41300082	0.265968362	−5.312665055	1.08E−07	3.17E−06
ENSG00000084676	NCOA1	484.1413512	1.803164225	0.216854668	8.315081421	9.17E−17	2.07E−14
ENSG00000070614	NDST1	886.1050176	−1.042070124	0.218972426	−4.758910252	1.95E−06	3.97E−05
ENSG00000184752	NDUFA12	349.4177146	1.275803454	0.269774632	4.729145382	2.25E−06	4.50E−05
ENSG00000189043	NDUFA4	2339.504529	1.061872319	0.150011955	7.078584607	1.46E−12	1.40E−10
ENSG00000165264	NDUFB6	298.0914574	1.197479832	0.194412214	6.159488679	7.30E−10	3.69E−08
ENSG00000165525	NEMF	710.7691948	1.922375861	0.216538195	8.877768024	6.82E−19	2.08E−16
ENSG00000102908	NFAT5	343.9702293	−1.418995082	0.281481585	−5.041164876	4.63E−07	1.14E−05
ENSG00000131196	NFATC1	278.5401271	−1.132910387	0.204585753	−5.537582032	3.07E−08	1.04E−06
ENSG00000165030	NFIL3	101.8190087	1.690415268	0.437963977	3.859713026	0.00011352	0.001296393
ENSG00000144802	NFKBIZ	429.7509153	−1.166942935	0.310526148	−3.75795386	0.000171308	0.001835185
ENSG00000140157	NIPA2	115.5966927	−1.202754156	0.357561328	−3.363770247	0.000768855	0.006324166
ENSG00000164190	NIPBL	244.7068754	1.383268976	0.271522316	5.094494615	3.50E−07	8.95E−06
ENSG00000114857	NKTR	265.4387786	1.089704593	0.223425844	4.877254015	1.08E−06	2.40E−05
ENSG00000087095	NLK	226.6383729	1.01550909	0.210180511	4.83160445	1.35E−06	2.92E−05
ENSG00000103202	NME4	910.4116755	−1.629079965	0.285069935	−5.714667749	1.10E−08	4.25E−07
ENSG00000123609	NMI	448.3147508	1.350494368	0.216946891	6.224999874	4.82E−10	2.53E−08
ENSG00000105373	NOP53	337.2256671	−1.601075348	0.417747407	−3.832639819	0.000126776	0.001422753
ENSG00000156642	NPTN	1914.068426	−1.70301525	0.202860079	−8.395024087	4.66E−17	1.12E−14
ENSG00000123358	NR4A1	115.7884017	−1.691520104	0.4546129	−3.720792135	0.000198599	0.002069902
ENSG00000148572	NRBF2	228.6218524	1.106920946	0.268510094	4.122455625	3.75E−05	0.000503997
ENSG00000134986	NREP	213.2553014	−1.358839634	0.221942231	−6.122492457	9.21E−10	4.47E−08
ENSG00000154146	NRGN	18927.13313	−1.48524345	0.259960292	−5.713347371	1.11E−08	4.28E−07
ENSG00000185115	NSMCE3	277.2197317	−1.176940776	0.287084531	−4.09963146	4.14E−05	0.000549298
ENSG00000157045	NTAN1	378.1388009	−1.208159939	0.19459668	−6.208533149	5.35E−10	2.77E−08
ENSG00000070081	NUCB2	357.5362432	1.223714492	0.179253708	6.826717866	8.69E−12	6.83E−10
ENSG00000069275	NUCKS1	210.0920681	1.589469176	0.233061126	6.819966937	9.11E−12	7.12E−10
ENSG00000213965	NUDT19	519.9095476	2.178754942	0.289464615	7.5268438	5.20E−14	6.55E−12
ENSG00000126883	NUP214	210.8607759	1.035685647	0.238030526	4.351062297	1.35E−05	0.000213736
ENSG00000093000	NUP50	300.0992239	1.313387613	0.253162688	5.187919364	2.13E−07	5.69E−06
ENSG00000147206	NXF3	350.0737351	2.835304533	0.441741229	6.418473866	1.38E−10	8.07E−09
ENSG00000135114	OASL	198.162174	1.400115167	0.360981558	3.878633508	0.000105045	0.001214301
ENSG00000115758	ODC1	6151.044272	−1.103709284	0.1333359	−8.277660289	1.26E−16	2.65E−14
ENSG00000060491	OGFR	127.5016577	−1.446184336	0.44718811	−3.233950776	0.001220905	0.009264092
ENSG00000238243	OR2W3	309.8218949	−1.423567349	0.318186302	−4.474005769	7.68E−06	0.000129695
ENSG00000249646	OR7E94P	100.5359947	2.165797343	0.249056986	8.695991147	3.44E−18	9.67E−16
ENSG00000172057	ORMDL3	115.4587103	1.221381798	0.220333903	5.543322111	2.97E−08	1.02E−06
ENSG00000184792	OSBP2	1132.859074	−1.162204806	0.260074699	−4.468734597	7.87E−06	0.000132727
ENSG00000181631	P2RY13	298.9071565	1.620442613	0.375792701	4.312065161	1.62E−05	0.000247159
ENSG00000168092	PAFAH1B2	687.2814541	1.737509844	0.278136233	6.246974102	4.18E−10	2.24E−08
ENSG00000152520	PAN3	1089.523785	2.281852812	0.291983832	7.814997142	5.50E−15	8.73E−13
ENSG00000182749	PAQR7	131.4435776	1.42134993	0.258630595	5.495675908	3.89E−08	1.28E−06
ENSG00000178685	PARP10	166.8841571	−1.422956703	0.522236473	−2.724736352	0.006435285	0.035172848
ENSG00000169564	PCBP1	477.1368213	−2.13507417	0.184935756	−11.54495059	7.83E−31	1.56E−27
ENSG00000184226	PCDH9	217.827159	1.386402338	0.238415477	5.815068532	6.06E−09	2.50E−07
ENSG00000247774	PCED1B-AS1	407.2233747	1.006545664	0.24184926	4.161872012	3.16E−05	0.000438927
ENSG00000141179	PCTP	334.7425245	1.035290323	0.245688353	4.213835583	2.51E−05	0.000360596
ENSG00000186642	PDE2A	152.4380874	−1.183898778	0.302175159	−3.917922253	8.93E−05	0.001055849
ENSG00000156973	PDE6D	225.6814926	1.002428501	0.23240505	4.313281928	1.61E−05	0.000246489
ENSG00000145431	PDGFC	740.5211031	1.695611448	0.220533443	7.688681687	1.49E−14	2.13E−12
ENSG00000083642	PDS5B	272.4528879	1.663085135	0.299484076	5.553167157	2.81E−08	9.69E−07
ENSG00000165650	PDZD8	109.0381114	1.226869087	0.372949361	3.289639869	0.001003157	0.007895989
ENSG00000162734	PEA15	334.9211419	−1.048045326	0.215985712	−4.852382675	1.22E−06	2.68E−05
ENSG00000179094	PER1	112.8613648	−2.025805223	0.385773111	−5.251286746	1.51E−07	4.26E−06
ENSG00000141959	PFKL	798.6219015	−1.21150229	0.304806382	−3.97466182	7.05E−05	0.000863303
ENSG00000255200	PGAM1P8	409.2533906	1.223801245	0.210464112	5.814774	6.07E−09	2.50E−07
ENSG00000164219	PGGT1B	160.4337536	1.167935835	0.344157755	3.393606041	0.000689788	0.00580444
ENSG00000112419	PHACTR2	508.1870488	1.5990193	0.251060272	6.369065429	1.90E−10	1.10E−08
ENSG00000112511	PHF1	111.0448303	−1.034150378	0.347702703	−2.974237386	0.002937178	0.018966244
ENSG00000175309	PHYKPL	283.0218244	−1.086828831	0.272619108	−3.986620149	6.70E−05	0.000826607
ENSG00000073921	PICALM	186.226772	1.225917283	0.269240832	4.553236864	5.28E−06	9.33E−05
ENSG00000145675	PIK3R1	229.4754968	−1.346495447	0.29051944	−4.634786053	3.57E−06	6.66E−05
ENSG00000141506	PIK3R5	238.3808342	−1.272498482	0.315577396	−4.032286527	5.52E−05	0.000701544
ENSG00000198355	PIM3	172.267231	−1.549806359	0.367643582	−4.215513164	2.49E−05	0.000359063
ENSG00000150867	PIP4K2A	28804.76291	2.353920968	0.231175405	10.18240225	2.38E−24	1.58E−21
ENSG00000186111	PIP5K1C	122.8017542	−1.150237014	0.281910622	−4.080147842	4.50E−05	0.000589615
ENSG00000154217	PITPNC1	140.3259853	−1.06018903	0.334673149	−3.167834146	0.001535791	0.011199988
ENSG00000171033	PKIA	516.5993902	−1.41589695	0.246657001	−5.740347706	9.45E−09	3.73E−07
ENSG00000011422	PLAUR	849.1829014	1.099769692	0.295462568	3.722196353	0.000197497	0.002061361
ENSG00000115896	PLCL1	218.2448471	1.124974799	0.273404235	4.114694128	3.88E−05	0.000519031
ENSG00000166428	PLD4	222.1361498	−1.305973447	0.372580825	−3.505208424	0.00045625	0.004121704
ENSG00000083444	PLOD1	190.1205698	−1.428559633	0.372441089	−3.835666034	0.000125224	0.001408952
ENSG00000198753	PLXNB3	134.403945	−1.870737184	0.290617058	−6.437121055	1.22E−10	7.20E−09
ENSG00000140464	PML	128.2514228	−1.27042212	0.3682391	−3.449992467	0.000560602	0.004875054
ENSG00000177666	PNPLA2	128.650058	−1.326714666	0.335995724	−3.948605799	7.86E−05	0.000947136
ENSG00000032444	PNPLA6	139.5408098	−1.140713966	0.311627888	−3.660500266	0.000251723	0.002507969
ENSG00000181222	POLR2A	441.9905295	−1.296533791	0.294199408	−4.406989805	1.05E−05	0.000170136
ENSG00000224897	POT1-AS1	116.6964165	2.113724469	0.323360016	6.536752729	6.29E−11	4.01E−09
ENSG00000188219	POTEE	308.4158519	−2.051239438	0.183406629	−11.1841074	4.88E−29	6.69E−26
ENSG00000125534	PPDPF	711.2236704	−2.447232307	0.299480094	−8.17160256	3.04E−16	5.96E−14
ENSG00000131626	PPFIA1	326.470843	1.278810227	0.203974884	6.269449458	3.62E−10	1.97E−08
ENSG00000084072	PPIE	682.1613686	1.03886024	0.175190526	5.929888234	3.03E−09	1.31E−07
ENSG00000213639	PPP1CB	5170.282644	2.679293503	0.267092705	10.03132416	1.11E−23	6.96E−21
ENSG00000113575	PPP2CA	430.6476764	−1.503043096	0.29065394	−5.171246237	2.33E−07	6.17E−06
ENSG00000104695	PPP2CB	100.4862499	2.068158024	0.301419401	6.861396509	6.82E−12	5.52E−10
ENSG00000138814	PPP3CA	1324.716783	2.193677465	0.280837638	7.811194676	5.66E−15	8.94E−13
ENSG00000120910	PPP3CC	124.0278907	−1.332025995	0.395098584	−3.371376278	0.000747936	0.006182244
ENSG00000149923	PPP4C	775.6016852	−1.021428117	0.302365174	−3.378127523	0.000729812	0.006059846
ENSG00000105063	PPP6R1	482.3964581	−1.176757654	0.300776071	−3.912404503	9.14E−05	0.001076791
ENSG00000110075	PPP6R3	371.1562046	1.350643912	0.231395396	5.8369524	5.32E−09	2.22E−07
ENSG00000133246	PRAM1	167.6187287	−1.512293814	0.400306828	−3.777836672	0.000158197	0.001714826
ENSG00000167815	PRDX2	204.4054042	1.081952329	0.246188496	4.394812692	1.11E−05	0.000178636
ENSG00000154229	PRKCA	442.1043967	1.663799631	0.249397169	6.671285141	2.54E−11	1.77E−09
ENSG00000163932	PRKCD	1873.087804	−1.027582242	0.179982898	−5.7093327	1.13E−08	4.37E−07
ENSG00000130175	PRKCSH	176.530105	−1.865197106	0.301983622	−6.176484314	6.55E−10	3.34E−08
ENSG00000120685	PROSER1	117.6895535	−1.167909426	0.348177233	−3.354353231	0.000795507	0.006494382
ENSG00000101911	PRPS2	175.6045094	1.927876559	0.335937109	5.738802023	9.53E−09	3.75E−07
ENSG00000131188	PRR7	476.8030066	−2.990151669	0.289694136	−10.32175421	5.62E−25	4.25E−22
ENSG00000257621	PSMA3-AS1	523.0731818	1.278041871	0.219936523	5.81095787	6.21E−09	2.55E−07
ENSG00000041357	PSMA4	471.555119	1.01636927	0.218156768	4.658894065	3.18E−06	6.04E−05
ENSG00000159377	PSMB4	705.2126907	1.18845387	0.227919142	5.214366197	1.84E−07	5.04E−06
ENSG00000225131	PSME2P2	189.7419679	1.011364405	0.205314451	4.925928987	8.40E−07	1.92E−05
ENSG00000068878	PSME4	121.8887447	1.060829038	0.308846031	3.434815189	0.000592958	0.005107218
ENSG00000011304	PTBP1	610.8844909	−1.348279065	0.239407435	−5.631734301	1.78E−08	6.51E−07
ENSG00000197744	PTMAP2	4022.965036	1.012981094	0.134006005	7.55922162	4.05E−14	5.26E−12
ENSG00000214182	PTMAP5	2543.76238	1.666124128	0.129188648	12.89683071	4.69E−38	3.43E−34
ENSG00000119383	PTPA	363.7562363	−1.123622997	0.261658581	−4.294233322	1.75E−05	0.000264382
ENSG00000127947	PTPN12	2451.447491	1.257468402	0.176083731	7.141309396	9.24E−13	9.21E−11
ENSG00000055917	PUM2	189.9463642	1.463622629	0.316264236	4.627847415	3.69E−06	6.84E−05
ENSG00000185129	PURA	105.3220621	1.049860022	0.22004552	4.771103817	1.83E−06	3.78E−05
ENSG00000179165	PXT1	138.1737437	1.889706462	0.224774064	8.407137514	4.20E−17	1.04E−14
ENSG00000115828	QPCT	278.8417115	1.746881578	0.369169536	4.731922351	2.22E−06	4.45E−05
ENSG00000179912	R3HDM2	276.7157562	1.205297399	0.254210897	4.741328606	2.12E−06	4.27E−05
ENSG00000198858	R3HDM4	4976.944581	−1.840683106	0.26204951	−7.024180687	2.15E−12	2.01E−10
ENSG00000185236	RAB11B	1243.663848	−1.573705007	0.266927625	−5.895624345	3.73E−09	1.59E−07
ENSG00000080371	RAB21	152.3340191	1.129783694	0.247599013	4.562957179	5.04E−06	8.95E−05
ENSG00000111540	RAB5B	662.5075087	−1.034230733	0.225831201	−4.579662728	4.66E−06	8.35E−05
ENSG00000125970	RALY	1448.956675	−1.469076561	0.264139888	−5.561736901	2.67E−08	9.28E−07
ENSG00000138698	RAP1GDS1	879.7949859	1.117714831	0.225959062	4.946536875	7.55E−07	1.76E−05
ENSG00000125249	RAP2A	115.3010181	1.673882275	0.274587945	6.09597873	1.09E−09	5.14E−08
ENSG00000181467	RAP2B	4949.032912	1.590227514	0.210685322	7.547879935	4.42E−14	5.67E−12
ENSG00000123728	RAP2C	212.3906813	1.629274625	0.290541304	5.607721179	2.05E−08	7.37E−07
ENSG00000185989	RASA3	2214.190863	−1.278060603	0.161863771	−7.895902785	2.88E−15	4.94E−13
ENSG00000105122	RASAL3	161.7870513	−1.298495459	0.41225099	−3.149769171	0.001633995	0.011802331
ENSG00000138670	RASGEF1B	129.0497252	1.479656455	0.362634865	4.080292873	4.50E−05	0.000589615
ENSG00000068831	RASGRP2	2210.40412	−1.035266361	0.173317417	−5.973239042	2.33E−09	1.02E−07
ENSG00000153179	RASSF3	246.3612569	1.413517483	0.294825303	4.794423918	1.63E−06	3.44E−05
ENSG00000166831	RBPMS2	433.0608897	−1.214853271	0.245092332	−4.956716757	7.17E−07	1.68E−05
ENSG00000183688	RFLNB	620.905646	1.400898879	0.202895195	6.904544375	5.04E−12	4.20E−10
ENSG00000159496	RGL4	111.4192497	1.409027131	0.302855977	4.652465978	3.28E−06	6.21E−05
ENSG00000171700	RGS19	670.4024207	−1.188485728	0.330420034	−3.596893669	0.00032204	0.003082504
ENSG00000116741	RGS2	2797.434683	1.189122982	0.252520497	4.709015679	2.49E−06	4.91E−05
ENSG00000187010	RHD	1395.963169	1.29508614	0.178900418	7.2391454	4.52E−13	4.73E−11
ENSG00000132669	RIN2	236.1610429	1.997084658	0.324355076	6.157093898	7.41E−10	3.72E−08
ENSG00000100599	RIN3	233.833182	−1.122960711	0.340832634	−3.294757007	0.000985068	0.007773142
ENSG00000143622	RIT1	2467.068866	1.378194079	0.177334711	7.771710728	7.74E−15	1.16E−12
ENSG00000153561	RMND5A	2458.503993	1.883665991	0.227351102	8.285273211	1.18E−16	2.56E−14
ENSG00000274012	RN7SL2	947.5011339	−1.851847541	0.451041715	−4.105712355	4.03E−05	0.000536669
ENSG00000169413	RNASE6	281.2988445	1.37461481	0.315886563	4.351609	1.35E−05	0.000213511
ENSG00000026297	RNASET2	329.3685292	−1.10717259	0.282362315	−3.921106081	8.81E−05	0.00104481
ENSG00000123091	RNF11	13260.28079	−1.260605946	0.127066716	−9.920819473	3.38E−23	2.00E−20
ENSG00000070423	RNF126	263.3213362	−2.457808942	0.456282231	−5.386597974	7.18E−08	2.22E−06
ENSG00000082996	RNF13	666.4121099	1.007098664	0.167559184	6.010405648	1.85E−09	8.25E−08
ENSG00000158717	RNF166	351.7522436	−1.728919146	0.371945032	−4.648318961	3.35E–06	6.32E−05
ENSG00000163961	RNF168	247.8847272	1.146888482	0.245972788	4.662664075	3.12E−06	5.95E−05
ENSG00000103549	RNF40	110.9088132	−1.277291796	0.411866133	−3.101230454	0.001927182	0.013562639
ENSG00000117748	RPA2	369.4359325	1.032764781	0.248326566	4.158897695	3.20E−05	0.000443275
ENSG00000240356	RPL23AP7	899.3692224	1.005260182	0.379159731	2.651284141	0.008018635	0.041462072
ENSG00000227063	RPL41P1	121.1604835	1.89774894	0.1595469	11.89461492	1.26E−32	3.08E−29
ENSG00000189343	RPS2P46	171.6532621	−1.410501358	0.267885885	−5.265306756	1.40E−07	3.97E−06
ENSG00000240342	RPS2P5	429.477078	−1.250937888	0.275018575	−4.548557818	5.40E−06	9.48E−05
ENSG00000124782	RREB1	136.5666043	−1.737004919	0.346034527	−5.019744516	5.17E−07	1.26E−05
ENSG00000187257	RSBN1L	117.0813521	1.389840986	0.243761071	5.701652776	1.19E−08	4.56E−07
ENSG00000048649	RSF1	382.8077265	1.438255273	0.19845531	7.24725015	4.25E−13	4.51E−11
ENSG00000172426	RSPH9	272.9261989	1.017448109	0.213823115	4.758363508	1.95E−06	3.98E−05
ENSG00000125744	RTN2	132.1536786	−1.188167606	0.247642086	−4.797922774	1.60E−06	3.38E−05
ENSG00000163191	S100A11	2794.409357	1.086782818	0.215954028	5.032473011	4.84E−07	1.19E−05
ENSG00000167100	SAMD14	333.4310016	−1.581726681	0.277586424	−5.698141339	1.21E−08	4.63E−07
ENSG00000205413	SAMD9	141.8250128	1.084397869	0.304209562	3.564640972	0.000364355	0.003434689
ENSG00000177409	SAMD9L	149.9847943	1.022537938	0.291442814	3.508537138	0.000450578	0.004088015
ENSG00000150459	SAP18	1411.340748	1.026137005	0.159021085	6.452836152	1.10E−10	6.56E−09
ENSG00000079332	SAR1A	701.5048561	1.086122929	0.23739377	4.575195595	4.76E−06	8.50E−05
ENSG00000139218	SCAF11	649.9154956	1.078679715	0.218803531	4.929900852	8.23E−07	1.89E−05
ENSG00000188076	SCGB1C1	282.3249545	1.229679884	0.159204408	7.723906021	1.13E−14	1.66E−12
ENSG00000151466	SCLT1	326.0716495	1.991921905	0.213458919	9.331640552	1.04E−20	4.23E−18
ENSG00000105711	SCN1B	1991.673515	−1.235954568	0.25341617	−4.877173265	1.08E−06	2.40E−05
ENSG00000054282	SDCCAG8	351.1264071	1.102980796	0.205152837	5.376385767	7.60E−08	2.33E−06
ENSG00000078808	SDF4	265.0110196	−1.397175855	0.390192406	−3.580735637	0.000342628	0.003251187
ENSG00000265808	SEC22B	728.3501571	1.996979298	0.264114005	7.561050372	4.00E−14	5.22E−12
ENSG00000058262	SEC61A1	357.6361479	−1.071823031	0.304106607	−3.524497675	0.000424287	0.003894814
ENSG00000143416	SELENBP1	147.3277744	1.589191434	0.473174975	3.358570334	0.000783468	0.006413089
ENSG00000188404	SELL	3321.248021	1.287583757	0.221736775	5.806811958	6.37E−09	2.61E−07
ENSG00000127922	SEM1	244.611457	1.042719079	0.184208365	5.660541422	1.51E−08	5.66E−07
ENSG00000179918	SEPHS2	139.1134038	−1.016040414	0.336564035	−3.018862118	0.00253726	0.016906343
ENSG00000180096	SEPT1	291.8977368	−1.034046729	0.279987601	−3.693187567	0.000221461	0.002260898
ENSG00000184702	SEPT5	4582.423396	−1.737621061	0.279843799	−6.209253399	5.32E−10	2.77E−08
ENSG00000164300	SERINC5	235.3210348	2.104672759	0.421111328	4.997901076	5.80E−07	1.40E−05
ENSG00000119335	SET	4494.036227	2.637407793	0.244649218	10.78036469	4.26E−27	4.45E−24
ENSG00000168066	SF1	481.9038433	−1.833257301	0.257916353	−7.107952933	1.18E−12	1.15E−10
ENSG00000099995	SF3A1	430.8929088	−1.034057251	0.20945143	−4.93697872	7.93E−07	1.84E−05
ENSG00000104897	SF3A2	188.9317273	−1.969627146	0.357127046	−5.515200172	3.48E−08	1.16E−06
ENSG00000198053	SIRPA	104.6134148	−1.375874436	0.395295023	−3.480626758	0.000500242	0.00443942
ENSG00000182628	SKA2	460.8821235	1.356141222	0.207686508	6.529751176	6.59E−11	4.16E−09
ENSG00000136603	SKIL	150.5101729	1.738156717	0.334826892	5.191210024	2.09E−07	5.62E−06
ENSG00000147454	SLC25A37	2816.560457	2.335221639	0.31224376	7.478841659	7.50E−14	9.24E−12
ENSG00000077713	SLC25A43	172.4161029	−1.858321495	0.380807845	−4.87994541	1.06E−06	2.37E−05
ENSG00000145740	SLC30A5	135.6382586	−1.053755654	0.272241808	−3.870660645	0.000108541	0.001248789
ENSG00000162695	SLC30A7	101.0035081	1.066496345	0.391799966	2.722042978	0.00648797	0.035356537
ENSG00000189339	SLC35E2B	113.7808838	−1.263154298	0.288911408	−4.37211638	1.23E−05	0.000196527
ENSG00000111371	SLC38A1	567.8208161	−1.056860094	0.367337384	−2.877082867	0.004013702	0.024300515
ENSG00000129353	SLC44A2	5604.665253	−1.107063871	0.157534036	−7.027458316	2.10E−12	1.97E−10
ENSG00000004939	SLC4A1	116.4350858	1.911290759	0.456958386	4.18263636	2.88E−05	0.000405311
ENSG00000090020	SLC9A1	207.9929708	−1.322106074	0.279280416	−4.733973454	2.20E−06	4.41E−05
ENSG00000113810	SMC4	103.4641112	1.661203118	0.241172026	6.888042306	5.66E−12	4.63E−10
ENSG00000198952	SMG5	146.7563922	−1.319690913	0.316358577	−4.171503507	3.03E−05	0.000422998
ENSG00000223553	SMPD4P1	264.1332131	1.26494931	0.410242714	3.083416883	0.002046383	0.014237032
ENSG00000099940	SNAP29	678.4170536	−1.395068577	0.205859477	−6.776800344	1.23E−11	9.52E−10
ENSG00000184602	SNN	12826.0602	−1.164800768	0.1582619	−7.35995693	1.84E−13	2.16E−11
ENSG00000104852	SNRNP70	116.076593	−1.597050104	0.413264479	−3.864474652	0.000111329	0.001274177
ENSG00000143376	SNX27	213.1043154	1.17393329	0.323342717	3.630616146	0.000282745	0.002765533
ENSG00000057252	SOAT1	107.2899431	1.357830653	0.393007439	3.454974429	0.000550345	0.004806259
ENSG00000184557	SOCS3	114.0236727	−1.11050898	0.422589138	−2.627869198	0.008592155	0.043716325
ENSG00000118363	SPCS2	235.1301672	1.145123432	0.20262892	5.651332643	1.59E−08	5.91E−07
ENSG00000228589	SPCS2P4	180.1655035	1.382335798	0.22230367	6.218232017	5.03E−10	2.63E−08
ENSG00000090487	SPG21	291.6750042	−1.017823404	0.257404816	−3.954173891	7.68E−05	0.000929436
ENSG00000176170	SPHK1	1049.990141	−1.501127529	0.247701243	−6.060234129	1.36E−09	6.24E−08
ENSG00000066336	SPI1	341.2101652	−2.016546753	0.338913566	−5.950032568	2.68E−09	1.16E−07
ENSG00000197122	SRC	603.5590811	−1.136688103	0.236674319	−4.802752181	1.56E−06	3.31E−05
ENSG00000198911	SREBF2	180.9524504	−1.530822277	0.274016746	−5.586601176	2.32E−08	8.24E−07
ENSG00000100883	SRP54	104.7764717	1.155484963	0.322049379	3.587912409	0.000333336	0.003174004
ENSG00000124193	SRSF6	12006.81228	1.325907756	0.246378854	5.38158098	7.38E−08	2.27E−06
ENSG00000157216	SSBP3	110.5239844	1.153110493	0.209705266	5.498719787	3.83E−08	1.26E−06
ENSG00000113532	ST8SIA4	176.0246283	1.217852246	0.299651707	4.064225959	4.82E−05	0.000623116
ENSG00000040341	STAU2	923.6671427	1.080064123	0.1914179	5.642440567	1.68E−08	6.17E−07
ENSG00000081320	STK17B	1730.25007	1.31998086	0.284800213	4.634760792	3.57E−06	6.66E−05
ENSG00000196182	STK40	1563.931331	−1.16156106	0.198751776	−5.844280159	5.09E−09	2.13E−07
ENSG00000140022	STON2	3022.456346	1.598390977	0.20739099	7.707137972	1.29E−14	1.88E−12
ENSG00000196792	STRN3	195.9013003	2.865460515	0.300414452	9.538357752	1.45E−21	6.94E−19
ENSG00000103266	STUB1	164.5731268	−1.415407853	0.369081232	−3.834949409	0.00012559	0.001410895
ENSG00000165416	SUGT1	149.0514546	1.007475479	0.260551483	3.866704068	0.000110316	0.001265294
ENSG00000116030	SUMO1	618.5466033	1.08865026	0.18516112	5.879475457	4.12E−09	1.75E−07
ENSG00000235082	SUMO1P3	113.746363	1.088954432	0.179034515	6.082371506	1.18E−09	5.55E−08
ENSG00000100242	SUN2	214.8496765	−1.481206553	0.372931177	−3.97179599	7.13E−05	0.000871452
ENSG00000106868	SUSD1	1268.109844	−1.166172708	0.164780825	−7.07711415	1.47E−12	1.41E-10
ENSG00000117614	SYF2	477.5295184	1.139252049	0.205289219	5.549497704	2.86E−08	9.88E−07
ENSG00000125755	SYMPK	179.7283908	−1.392017534	0.249566383	−5.577744545	2.44E−08	8.63E−07
ENSG00000182253	SYNM	166.1479999	1.636361634	0.238854869	6.850861543	7.34E−12	5.92E−10
ENSG00000142765	SYTL1	239.6269306	−1.022238984	0.387458658	−2.638317567	0.008331852	0.042629888
ENSG00000055208	TAB2	412.9658464	1.267982858	0.223905727	5.663021109	1.49E−08	5.58E−07
ENSG00000165632	TAF3	128.5884155	1.126637787	0.283838971	3.969285058	7.21E−05	0.000879216
ENSG00000253676	TAGLN2P1	1645.996948	−1.693406048	0.177543063	−9.538001772	1.46E−21	6.94E−19
ENSG00000203705	TATDN3	111.8618888	1.001150508	0.276346821	3.622804503	0.000291426	0.002837781
ENSG00000104946	TBC1D17	140.3426965	−1.135221823	0.326959737	−3.47205388	0.000516493	0.004570318
ENSG00000177565	TBL1XR1	1650.189997	1.700323708	0.211576142	8.03646238	9.25E−16	1.69E−13
ENSG00000113649	TCERG1	124.3349673	−1.020379587	0.363838493	−2.804484972	0.005039704	0.029198326
ENSG00000110719	TCIRG1	611.7283154	−1.448733576	0.37586505	−3.854398207	0.000116015	0.001320916
ENSG00000111802	TDP2	443.7912564	1.513104519	0.211097212	7.167809097	7.62E−13	7.74E−11
ENSG00000088992	TESC	142.6923204	−1.070341411	0.30890541	−3.464948741	0.000530333	0.004663654
ENSG00000151575	TEX9	202.8562532	1.174906901	0.223625258	5.253909638	1.49E−07	4.21E−06
ENSG00000068323	TFE3	168.1610449	−1.319406058	0.248499752	−5.30948641	1.10E−07	3.21E−06
ENSG00000105329	TGFB1	884.3447709	−1.943526176	0.321876634	−6.038108922	1.56E−09	6.99E−08
ENSG00000069702	TGFBR3	115.4950655	−1.217044835	0.410940227	−2.961610361	0.003060348	0.019577121
ENSG00000177683	THAP5	318.4296409	2.394516689	0.327598949	7.309292948	2.69E−13	2.97E−11
ENSG00000066654	THUMPD1	641.2467368	1.102550851	0.223757137	4.927444396	8.33E−07	1.91E−05
ENSG00000151923	TIAL1	131.2180452	1.806682384	0.327691937	5.513356239	3.52E−08	1.18E−06
ENSG00000150779	TIMM8B	422.1106649	1.11605422	0.163009295	6.846567998	7.56E−12	6.08E−10
ENSG00000149476	TKFC	182.9265325	−1.446590535	0.289086307	−5.004009181	5.62E−07	1.36E−05
ENSG00000140332	TLE3	280.7292137	1.412666728	0.30726224	4.597593019	4.27E−06	7.75E−05
ENSG00000198586	TLK1	21134.4362	2.235491533	0.235591317	9.488853685	2.34E−21	1.07E−18
ENSG00000064115	TM7SF3	286.9703275	−1.167712513	0.262779113	−4.443703678	8.84E−06	0.000146897
ENSG00000167895	TMC8	238.8610013	−1.423579517	0.371760909	−3.829287805	0.000128515	0.001437856
ENSG00000143183	TMCO1	209.0536831	1.01529547	0.264745353	3.834988828	0.00012557	0.001410895
ENSG00000168936	TMEM129	148.4281418	−1.043353267	0.359608724	−2.901356944	0.003715504	0.022874027
ENSG00000170006	TMEM154	531.9240031	1.296472928	0.281393065	4.607337889	4.08E−06	7.45E−05
ENSG00000215712	TMEM242	107.8371407	1.240237642	0.266096219	4.660861582	3.15E−06	5.99E−05
ENSG00000106771	TMEM245	130.3589951	1.277477702	0.336073669	3.80118355	0.000144007	0.001584506
ENSG00000106609	TMEM248	517.7223755	−1.009216116	0.199845694	−5.049976779	4.42E−07	1.10E−05
ENSG00000109133	TMEM33	307.6901766	2.376312117	0.33217615	7.153771019	8.44E−13	8.53E−11
ENSG00000166471	TMEM41B	131.6019293	1.558171653	0.294255254	5.295306143	1.19E−07	3.45E−06
ENSG00000116209	TMEM59	1373.881769	1.052531918	0.166608622	6.317391672	2.66E−10	1.48E−08
ENSG00000165071	TMEM71	403.5143325	1.350643552	0.214366827	6.300618292	2.96E−10	1.63E−08
ENSG00000144747	TMF1	129.2075192	1.00027042	0.27244188	3.671500216	0.000241131	0.002427805
ENSG00000164897	TMUB1	150.5706264	−1.172097129	0.338286463	−3.464806482	0.000530613	0.004663654
ENSG00000118503	TNFAIP3	486.9996203	−1.531186631	0.339859726	−4.505348868	6.63E−06	0.000113535
ENSG00000163154	TNFAIP8L2	118.4693015	1.595654016	0.317563833	5.024671742	5.04E−07	1.23E−05
ENSG00000028137	TNFRSF1B	1020.027909	−1.208487741	0.311232376	−3.882911397	0.000103213	0.00119628
ENSG00000121858	TNFSF10	442.237252	1.509007856	0.33877659	4.454286098	8.42E−06	0.000140688
ENSG00000145901	TNIP1	285.3538767	−1.420884215	0.251602273	−5.647342518	1.63E−08	6.02E−07
ENSG00000100354	TNRC6B	584.344869	1.787614809	0.273412381	6.538163362	6.23E−11	3.98E−09
ENSG00000141232	TOB1	544.7191725	1.324425846	0.340273922	3.89223435	9.93E−05	0.001157334
ENSG00000198900	TOP1	297.4446063	1.086052596	0.147613962	7.35738398	1.88E−13	2.19E−11
ENSG00000077097	TOP2B	158.6159687	1.177825009	0.273205353	4.311134451	1.62E−05	0.000247856
ENSG00000169905	TOR1AIP2	248.3372312	1.243617793	0.242609016	5.126016395	2.96E−07	7.72E−06
ENSG00000164548	TRA2A	545.8634327	1.317347663	0.232803138	5.658633618	1.53E−08	5.71E−07
ENSG00000170638	TRABD	165.2517581	−1.158020144	0.395944592	−2.924702518	0.003447856	0.021569954
ENSG00000054116	TRAPPC3	295.2608408	1.16015881	0.224461202	5.168638486	2.36E−07	6.25E−06
ENSG00000167632	TRAPPC9	582.0213076	−1.26049879	0.235586105	−5.350480204	8.77E−08	2.66E−06
ENSG00000163519	TRAT1	104.4117193	2.276398751	0.468651362	4.857339459	1.19E−06	2.62E−05
ENSG00000124731	TREM1	909.4005682	1.126058821	0.265259813	4.245116554	2.18E−05	0.000321116
ENSG00000281103	TRG-AS1	213.5834504	1.267952897	0.229816867	5.517231672	3.44E−08	1.15E−06
ENSG00000204977	TRIM13	378.4346071	1.32430348	0.203633007	6.503383211	7.85E−11	4.82E−09
ENSG00000162722	TRIM58	4990.833662	−1.474498097	0.146137517	−10.08979846	6.13E−24	3.95E−21
ENSG00000171206	TRIM8	224.7505303	−1.00603083	0.266863427	−3.769834036	0.000163356	0.001762045
ENSG00000100815	TRIP11	142.5572981	1.626612881	0.233598527	6.96328399	3.32E−12	2.94E−10
ENSG00000100991	TRPC4AP	195.2270345	−1.479394475	0.352855967	−4.192629896	2.76E−05	0.000390865
ENSG00000166925	TSC22D4	454.8023094	−1.600337385	0.279840646	−5.71874532	1.07E−08	4.17E−07
ENSG00000265148	TSPOAP1-AS1	310.0520461	−1.282206624	0.191576311	−6.692928885	2.19E−11	1.56E−09
ENSG00000189241	TSPYL1	1652.185137	2.654852185	0.288380989	9.206058257	3.38E−20	1.30E−17
ENSG00000113312	TTC1	245.1779409	1.046111805	0.226110765	4.626545783	3.72E−06	6.87E−05
ENSG00000253352	TUG1	232.6771138	1.52897934	0.240590181	6.355119467	2.08E−10	1.19E−08
ENSG00000117862	TXNDC12	416.6246264	1.489560131	0.254419412	5.854742446	4.78E−09	2.01E−07
ENSG00000105397	TYK2	188.5202652	−1.137512579	0.353368963	−3.219050622	0.001286158	0.009665577
ENSG00000025708	TYMP	722.0453244	−1.147989793	0.372059393	−3.085501436	0.002032092	0.014160075
ENSG00000163714	U2SURP	257.508112	1.25243092	0.291538137	4.295941975	1.74E−05	0.000262896
ENSG00000137831	UACA	145.9375547	1.220227637	0.29251798	4.171462	3.03E−05	0.000422998
ENSG00000119048	UBE2B	1096.701117	1.694279274	0.192675014	8.793456074	1.45E−18	4.30E−16
ENSG00000186591	UBE2H	1062.120948	1.102282149	0.177685543	6.203555626	5.52E−10	2.84E−08
ENSG00000198833	UBE2J1	1443.708531	−1.167034321	0.155221225	−7.518522813	5.54E−14	6.94E−12
ENSG00000160714	UBE2Q1	176.3556485	1.47327986	0.225965317	6.519938025	7.03E−11	4.37E−09
ENSG00000140474	ULK3	155.7443267	−1.274176484	0.385764778	−3.302988136	0.000956604	0.007598219
ENSG00000092929	UNC13D	1668.279246	−1.200747091	0.248878053	−4.824640319	1.40E−06	3.00E−05
ENSG00000184076	UQCR10	1431.026237	1.026991018	0.148797769	6.901924865	5.13E−12	4.25E−10
ENSG00000156467	UQCRB	272.1298958	1.059670965	0.161316452	6.568895808	5.07E−11	3.31E−09
ENSG00000105698	USF2	876.8852982	1.229875668	0.163702932	7.512850589	5.79E−14	7.21E−12
ENSG00000162607	USP1	206.9564817	1.099142218	0.246604401	4.457107074	8.31E−06	0.000138956
ENSG00000103404	USP31	144.2421525	1.199373732	0.237831225	5.042961592	4.58E−07	1.13E−05
ENSG00000187555	USP7	131.4892498	1.360170082	0.294264537	4.622269792	3.80E−06	6.99E−05
ENSG00000138592	USP8	161.0492057	1.146442457	0.273761967	4.187734578	2.82E−05	0.000397846
ENSG00000125753	VASP	514.6602655	−1.113246984	0.235611846	−4.724919415	2.30E−06	4.58E−05
ENSG00000108828	VAT1	103.6923341	−1.467551263	0.383741431	−3.824323214	0.000131132	0.00146266
ENSG00000229124	VIM-AS1	5228.36279	1.243275723	0.171033364	7.269199946	3.62E−13	3.93E−11
ENSG00000062716	VMP1	1215.411207	1.495397625	0.232430607	6.433737991	1.25E−10	7.32E−09
ENSG00000112303	VNN2	394.1437111	1.155606998	0.304115019	3.799901108	0.000144754	0.001591131
ENSG00000093134	VNN3	105.1864776	1.775974143	0.475142021	3.737775373	0.000185656	0.001963074
ENSG00000128218	VPREB3	100.9223747	1.110634341	0.330779843	3.357624004	0.000786155	0.006432486
ENSG00000139722	VPS37B	335.389767	−1.101195023	0.232859971	−4.729000935	2.26E−06	4.50E−05
ENSG00000149823	VPS51	116.5985915	−1.442573239	0.407552699	−3.539599281	0.000400735	0.003706334
ENSG00000166272	WBP1L	430.2585095	1.394981464	0.204260638	6.829418915	8.53E−12	6.73E−10
ENSG00000120688	WBP4	132.7329077	1.005356757	0.203222106	4.947083635	7.53E−07	1.76E−05
ENSG00000128815	WDFY4	103.0772065	−1.622381642	0.400203739	−4.053889268	5.04E−05	0.000647877
ENSG00000124116	WFDC3	104.2802037	1.126068612	0.244899568	4.598083288	4.26E−06	7.74E−05
ENSG00000176871	WSB2	283.0928019	1.395451835	0.19317354	7.223824942	5.05E−13	5.20E−11
ENSG00000047644	WWC3	161.1403764	1.217471849	0.226809642	5.36781347	7.97E−08	2.44E−06
ENSG00000100219	XBP1	372.9884417	−1.190309972	0.377744027	−3.15110203	0.001626557	0.011773547
ENSG00000145817	YIPF5	145.9981505	1.117444305	0.269076326	4.152889708	3.28E−05	0.000452221
ENSG00000185728	YTHDF3	210.9565095	−1.111571321	0.29010539	−3.831612096	0.000127306	0.00142725
ENSG00000225528	Z82206.1	1337.741505	1.250914652	0.177498705	7.047457894	1.82E−12	1.72E−10
ENSG00000115085	ZAP70	474.2247272	−1.285152726	0.369394548	−3.479078764	0.000503141	0.004457928
ENSG00000123200	ZC3H13	240.3451507	1.139049412	0.258181007	4.411824957	1.03E−05	0.000167246
ENSG00000105939	ZC3HAV1	713.211886	1.043960848	0.245254173	4.256648664	2.08E−05	0.00030623
ENSG00000104219	ZDHHC2	264.9675236	1.635347453	0.24936615	6.558016998	5.45E−11	3.55E−09
ENSG00000153786	ZDHHC7	211.0007768	−1.104988844	0.271408508	−4.071312477	4.67E−05	0.000609126
ENSG00000160445	ZER1	345.7514337	−1.79743157	0.289901195	−6.200152325	5.64E−10	2.90E−08
ENSG00000156639	ZFAND3	448.0229414	−1.749680305	0.194296926	−9.00518779	2.15E−19	7.05E−17
ENSG00000128016	ZFP36	2011.495707	−1.255245919	0.27176632	−4.618842823	3.86E−06	7.09E−05
ENSG00000152518	ZFP36L2	1263.270459	−1.813512795	0.300075631	−6.04351905	1.51E−09	6.79E−08
ENSG00000165512	ZNF22	147.1253423	1.07368024	0.272460376	3.94068398	8.12E−05	0.000974684
ENSG00000151789	ZNF385D	888.8663269	1.250999314	0.163137864	7.668356589	1.74E−14	2.48E−12
ENSG00000171443	ZNF524	116.9438002	−1.124624581	0.30415974	−3.697480088	0.00021775	0.002229247
ENSG00000213015	ZNF580	127.2707744	−2.009348326	0.345050848	−5.823339773	5.77E−09	2.40E−07
ENSG00000159840	ZYX	6972.347248	−1.854009487	0.217520626	−8.523373263	1.55E−17	4.00E−15

**Table 3:** Sample details of two algorithms

103 samples included in the training cohort of the binary algorithm				
Sample NO.	Patients (*n* = 67)		NC (*n* = 36)	
	1	58	110	172
	3	63	112	
	5	66	113	
	7	68	114	
	9	70	116	
	10	72	117	
	11	73	118	
	15	74	125	
	17	75	127	
	19	81	129	
	21	82	131	
	22	83	132	
	24	84	133	
	25	85	139	
	29	87	140	
	30	88	143	
	32	89	144	
	34	90	145	
	35	91	146	
	36	92	147	
	39	93	148	
	40	94	149	
	41	96	150	
	42	97	151	
	43	98	152	
	45	99	153	
	46	100	156	
	47	101	157	
	49	104	159	
	50	106	161	
	51	108	164	
	52	109	166	
	53		167	
	54		170	
	57		171	

69 samples included in the validation cohort of the binary algorithm				
Sample NO.	Patients (*n* = 42)		NC (*n* = 27)	
	2	62	111	168
	4	64	115	169
	6	65	119	
	8	67	120	
	12	69	121	
	13	71	122	
	14	76	123	
	16	77	124	
	18	78	126	
	20	79	128	
	23	80	130	
	26	86	134	
	27	95	135	
	28	102	136	
	31	103	137	
	33	105	138	
	37	107	141	
	38		142	
	44		154	
	48		155	
	55		158	
	56		160	
	59		162	
	60		163	
	61		165	

103 samples included in the training cohort of the multiclass algorithm				
Sample NO.	TA (*n* = 20)	PTC (*n* = 26)	MPTC (*n* = 23)	NC (*n* = 34)
	2	33	76	111
	5	35	78	116
	6	36	80	119
	7	39	81	120
	8	40	82	122
	9	41	84	123
	10	42	85	125
	11	43	86	128
	14	44	88	129
	15	45	90	135
	18	50	92	136
	19	51	93	138
	21	52	94	140
	24	55	95	141
	25	59	98	142
	26	60	99	143
	27	61	101	145
	28	62	103	146
	31	63	104	147
	32	64	105	148
		65	107	149
		68	108	150
		70	109	151
		71		152
		72		156
		73		157
				159
				161
				162
				163
				166
				167
				169
				171

103 samples included in the training cohort of the multiclass algorithm				
Sample NO.	TA (*n* = 12)	PTC (*n* = 15)	MPTC (*n* = 13)	NC (*n* = 29)
	1	34	74	110
	3	37	75	112
	4	38	77	113
	12	46	79	114
	13	47	83	115
	16	48	87	117
	17	49	89	118
	20	53	91	121
	22	54	96	124
	23	56	97	126
	29	57	100	127
	30	58	102	130
		66	106	131
		67		132
		69		133
				134
				137
				139
				144
				153
				154
				155
				158
				160
				164
				165
				168
				170
				172

Sample NO. can be referred to Table S2

**Fig. 1:**
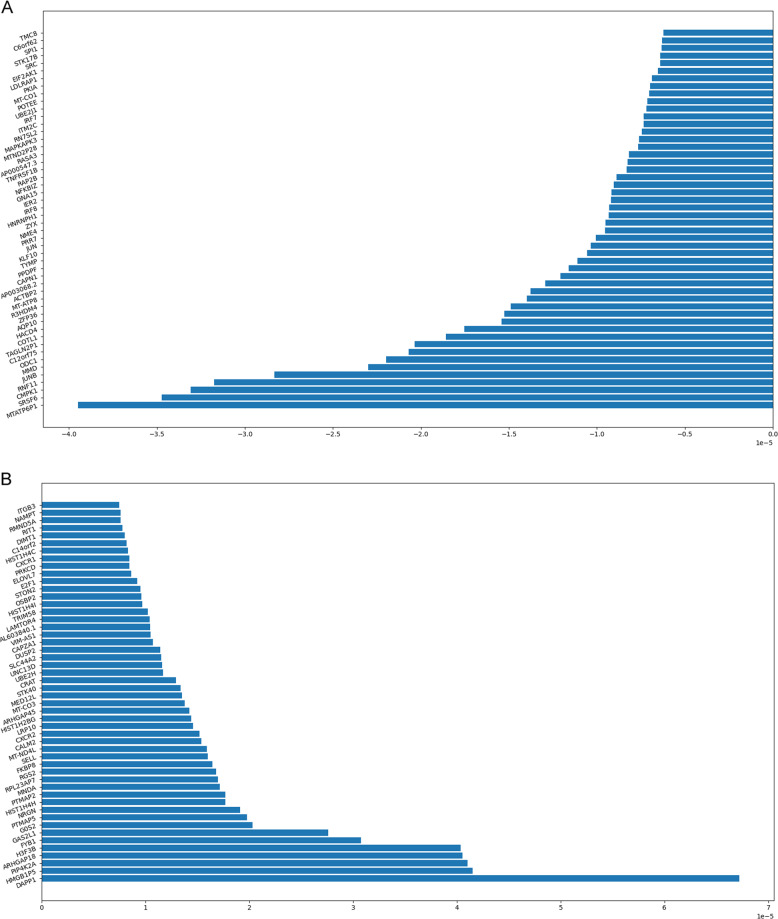
Weighted gene plot. The plot shows what features (genes) are being used by the binary algorithm to make classifications between healthy individuals and patients. Features (genes) presented on the bottom of the plot with longer blue lines are major contributors in the classification. **a** Genes on this plot contribute to classify healthy individuals. **b** Genes on this plot contribute to classify patients

**Fig. 2:**
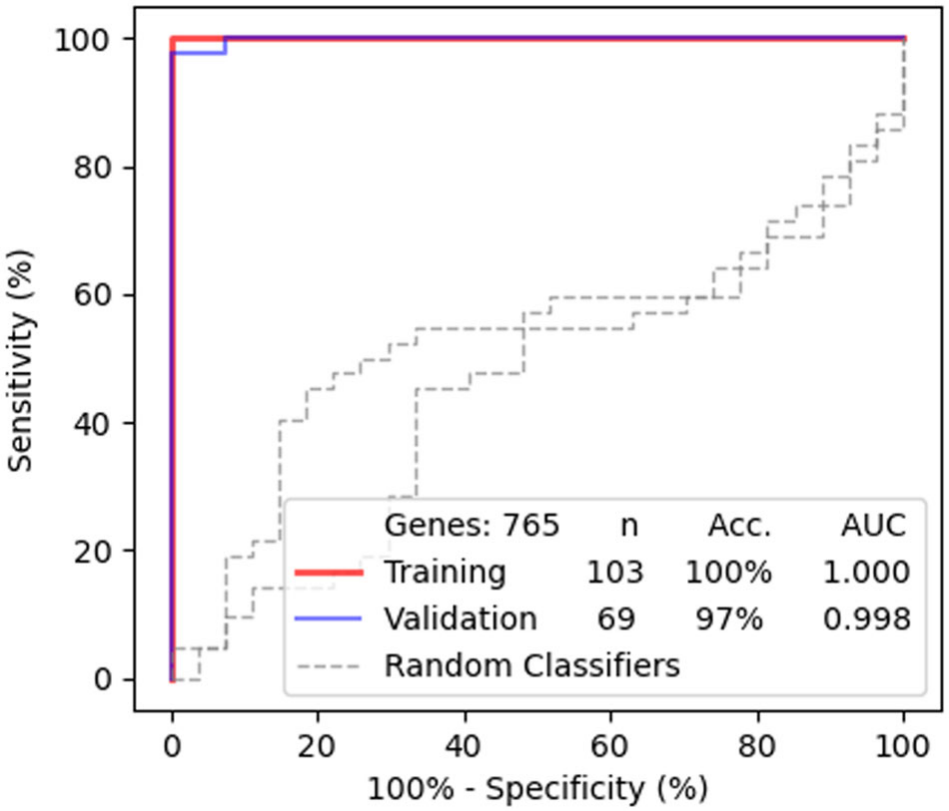
ROC-curve of SVM classifications. Red line indicated ROC evaluation of the training set (*n* = 103), blue line indicated ROC evaluation of the validation set (*n* = 69), gray dashed lines indicated the classification accuracies obtained by chance of the training and validation cohort. Indicated are cohort size, most optimal accuracy and AUC value

Next, we trained a binary diagnostic algorithm in a training set (*n* = 103). This algorithm employed the 765 GDTLs as classifier for sample classification, to which the contribution of each GDTL was evaluated at the meantime, yielding two top 50 weighted gene plots (Fig. 1). Subsequent validation employed a validation set (*n* = 69), yielding an accuracy of 97%, with an area under the curve (AUC) of 0.998 (Fig. 2). When the binary algorithm was executed 1000 times, we obtained an average accuracy of 96.5%. When the ratio of training:validation was changed from 60:40 to 40:60, the average accuracy decreased from 96.5% to 94.9%.

### Platelet RNA-seq allows for thyroid cancer diagnostics

**Fig. 3:**
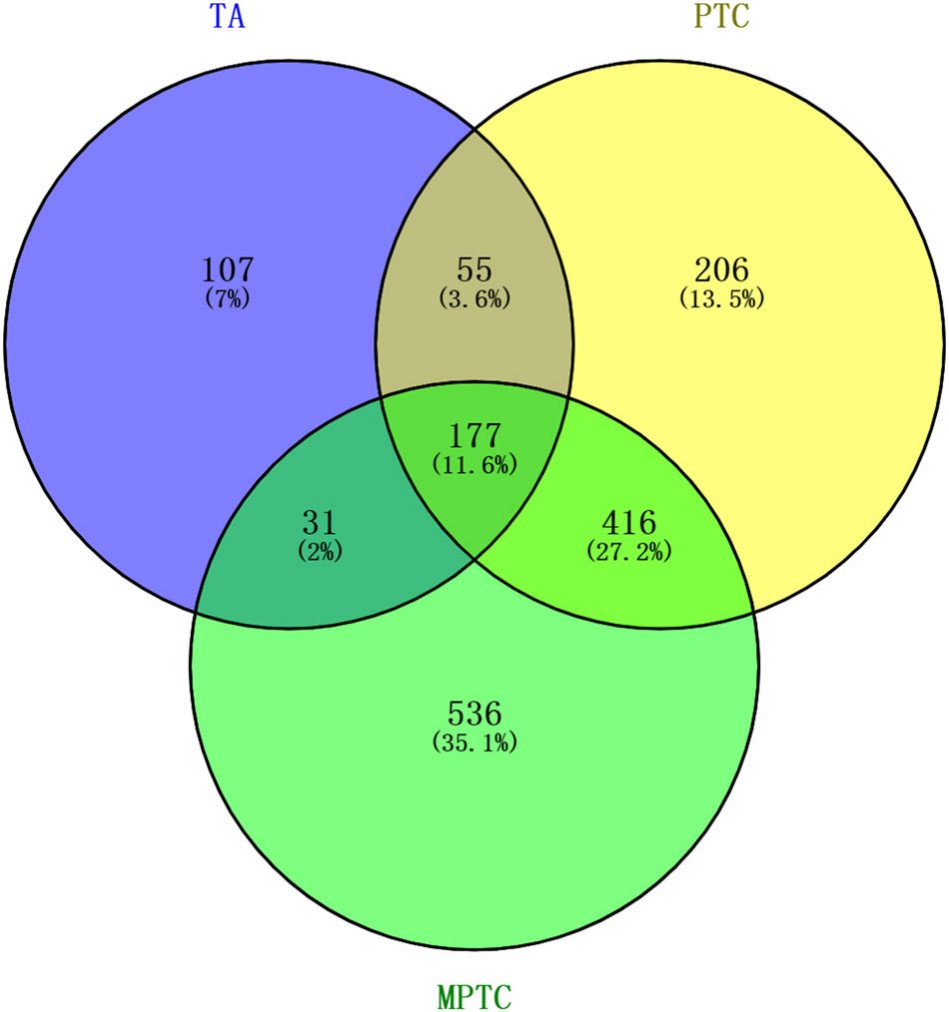
Venn analysis of DEGs. 370, 854 and 1160 DEGs were identified in platelets of healthy individuals compared to patients with TA, PTC, and MPTC, respectively. Among them, 107, 206, and 536 DEGs were included exclusively in their own classes. Different color areas represent different classes. The cross areas indicate co-differentially expressed genes

370, 854 and 1160 GDTLs were identified in platelets of healthy individuals compared to patients with TA (Table S3), PTC (Table S4) and MPTC (Table S5), respectively. We noticed significant increases in the number of GDTLs, suggesting that neoplasm types as well as neoplasm progression or metastasis had influence on the platelet transcriptome profile. To distinguish healthy individuals and patients with specific disease class, GDTLs included exclusively in the three classes were screened using VENNY 2.1 (Fig. 3), and this resulted in three class-specific gene lists (Table S6), which were used as the classifier for training and validation of the diagnostic algorithm.

**Fig. 4:**
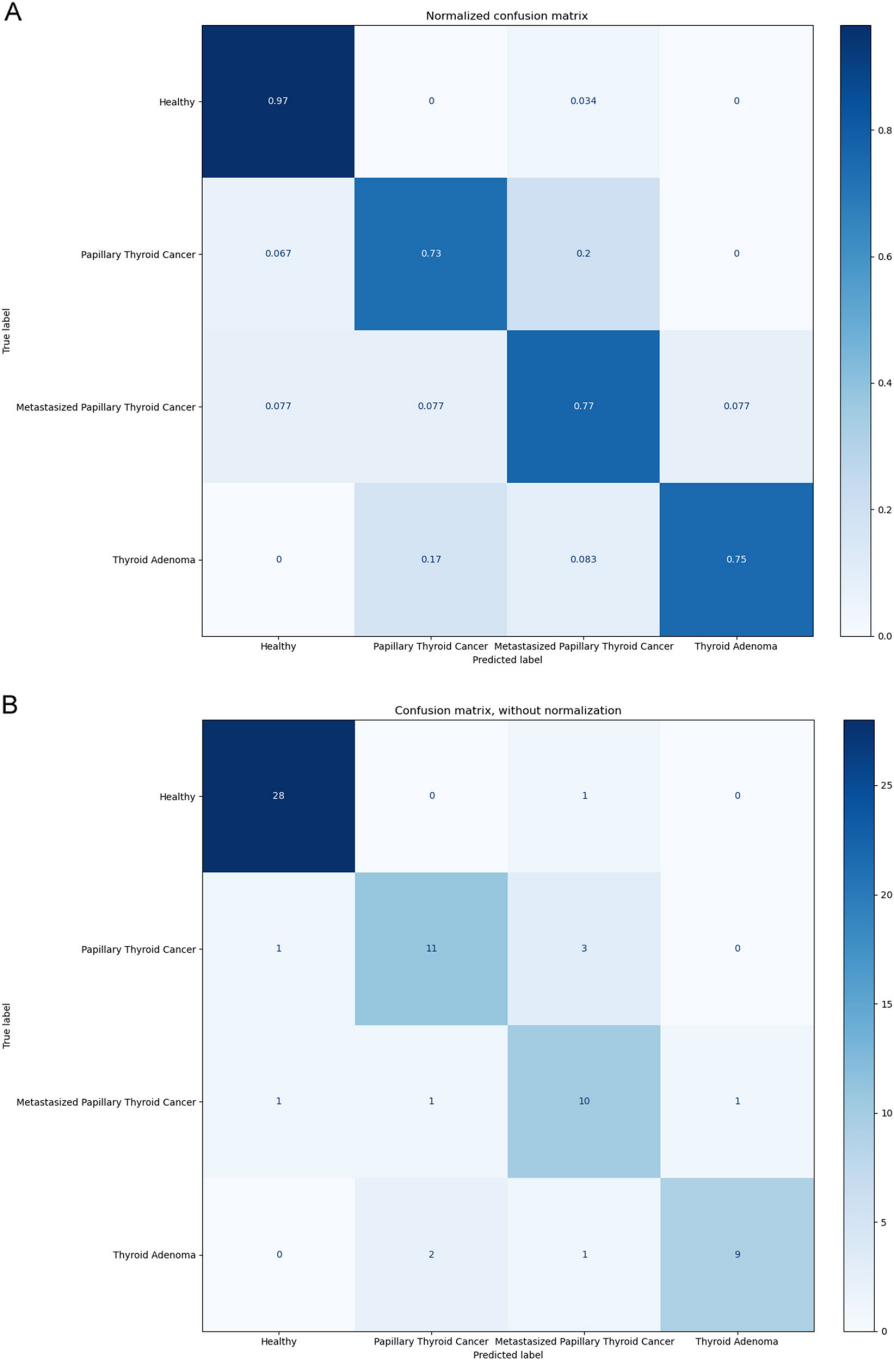
Confusion matrix of multiclass SVM algorithm in the validation cohort (*n* = 69) consisting of healthy individuals and patients with TA, PTC, and MPTC. Indicated are detection rates in percentages (**a**) and sample numbers (**b**)

The multiclass algorithm was also trained in a training set (*n* = 103), and we subsequently determined whether it could correctly classify each individual sample in the validation set (*n* = 69) as healthy individual or patient with one of the three neoplasms. The validation resulted in an average accuracy of 80.5% (Fig. 4), suggesting a thyroid neoplasm discriminative power of our algorithm. However, when we executed the multiclass algorithm 1000 times, an average accuracy of 63.9% was obtained.

### GO and KEGG enrichment analysis

Our date analysis yielded four GDTL lists (Tables 2, S3–S5). GO analysis revealed that most GDTLs were enriched for biological processes such as neutrophil degranulation, neutrophil activation, autophagy and regulation of multi-organism process (Fig. S1). KEGG pathway enrichment analysis demonstrated that GDTLs were mainly enriched in NOD-like receptor signaling pathway and pathways in endocytosis, osteoclast differentiation, human cytomegalovirus infection and tuberculosis (Fig. S2). *P*adj < 0.05 was used as the screening criterion.

## Discussion

We showed that two SVM algorithms that were developed based on platelet RNA-seq data could achieve high accuracy, sensitivity and specificity in thyroid neoplasm identification and also provide a strong indication on neoplasm type and metastasis. We also identified important genes and pathways in thyroid neoplasms using bioinformatics methods.

In our study, for the first time, the SVM algorithm and platelet RNA-seq were combined and employed for thyroid neoplasm diagnostics. Compared to similar studies that employed artificial intelligence system to develop diagnostic algorithms, the accuracy, AUC, sensitivity and specificity yielded by our algorithms were at levels similar to theirs. For example, also based on platelet mRNA profiles, Best et al. [9]. employed a leave-one-out cross-validation SVM algorithm to distinguish patients with neoplasms from healthy individuals, yielding an accuracy of 96%, with an AUC of 0.986. In Li et al.’s study [3], a deep convolutional neural network model was developed and trained based on 180668 images from 25235 controls and 131731 ultrasound images from 17627 patients with thyroid cancer, yielding a highest accuracy of 89.8%, with an AUC of 0.947. To test the stability of performances of our algorithms, we additionally executed both of them 1000 times, yielding an average accuracy of 96.5% for the binary algorithm and 63.9% for the algorithm. Compared to 97%, the accuracy we reported particularly, 96.5% suggests the performance of the binary algorithm was stable. When the ratio of training:validation was changed to 40:60, an accompanying decrease of average accuracy from 96.5% to 94.9% indicated a relatively high number of samples in the training set was needed to ensure a relatively accurate algorithm. By contrast, the performance of the multiclass algorithm seemed to be overestimated. After 1000 times execution, its overall accuracy declined to 63.9%, with accuracies of the four classes (NC, TA, PTC, MPTC) decreasing to 93.3%, 47.3%, 47.7% and 47.3%, respectively. This was probably due to transcript patterns of our samples are partially similar. When the training set comprised too many samples with similar transcript patterns, hyperplanes generated by the algorithm tended to separate these samples well but not classes, which resulted in the decreased accuracy. Although 80.5% seems to approach the upper limit of the multiclass algorithm’s accuracy, as long as the training set comprise more patient samples with distant transcript patterns, this high accuracy could be repeated, and the thyroid neoplasm discriminative power of the algorithm could also be proved.

Compared to recently published studies that used bioinformatics approaches to identify key genes and pathways in thyroid cancer, our study had a rather big number of samples (both patient and normal) analyzed. Based on four datasets (GSE3678, GSE3467, GSE33630 and GSE58545), Liang et al. [10] reported 225 differentially expressed genes, among which *PKIA*, *SELENBP1*, *CHI3L1*, *QPCT*, *KCNJ2*, *ITGA2* also showed to have differential transcript levels in our study. Other GDTLs we detected like *CXCR1*, *VASP*, *ZYX*, *GPR137* and *GOLPH3* were reported as known markers in many cancers [11–15].

Our study has some limitations. The two algorithms we presented were developed with a quiet small number of samples. The performance of both was believed to improved by including more samples, especially the multiclass algorithm’s. The binary algorithm was good at proving the capacity of a SVM algorithm in identifying thyroid neoplasm patient but not clinically practical so far, unless other diseases that share similar clinical symptoms with thyroid neoplasms are included. Besides, the SVM hyper parameter Cost-C was not fine-tuned. In principle, a bigger or a smaller C parameter results in a hyperplane with larger minimum margin or a hyperplane that correctly separate more training points, respectively. Because we used a default C parameter, the accuracy, sensitivity and specificity that our algorithms achieved might not be optimal. Also, we did not estimate the contribution of variables, such as patient age, gender, blood sample storage time, alcohol, smoking and treatment history to the altered platelet profile.

The capacity of SVM algorithms developed based on platelet RNA-seq data in identifying thyroid neoplasm patients and differentiating patients with multiclass thyroid neoplasms was proven in this study. Further algorithm development is warranted to include significantly more matched patient/healthy individual blood samples. Further validation is expected to determine the potential of our algorithms for therapy selection, disease recurrence monitoring, etc. Also, future studies should address causes and anticipated risks of samples misclassified in our study, such as cancer patients classified as healthy individuals.

## Supplementary information

Supplementary Figure S1

Supplementary Figure S2

Supplementary Table S1

Supplementary Table S2

Supplementary Table S3

Supplementary Table S4

Supplementary Table S5

Supplementary Table S6
