# Supplementary figures and images for: Diagnosis of thyroid neoplasm using support vector machine algorithms based on platelet RNA-seq
Source: Endocrine. 2020 Nov 12;72(3):758–83. doi: 10.1007/s12020-020-02523-x (PMC8159845; doi:10.1007/s12020-020-02523-x)

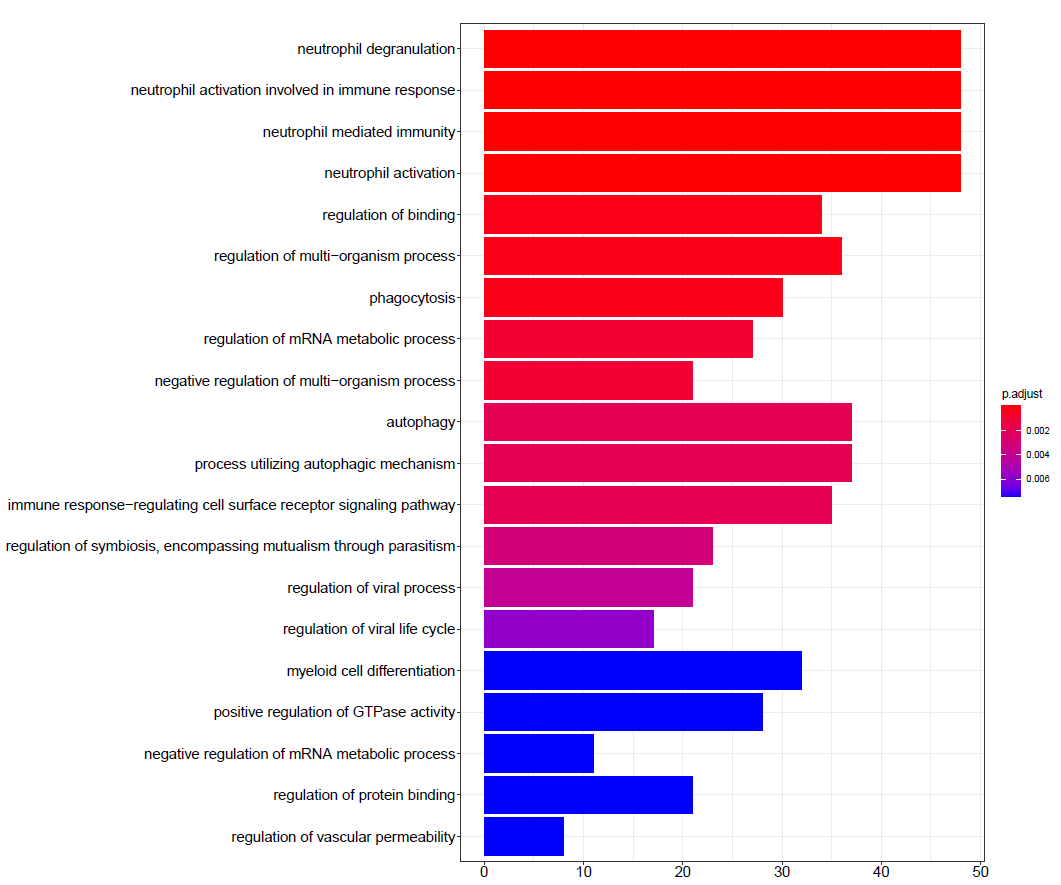


Fig. S1 Gene ontology analysis of GDTLs.

Supplement: Supplementary file 1 — Supplementary Figure S1 [file 12020_2020_2523_MOESM1_ESM.docx]

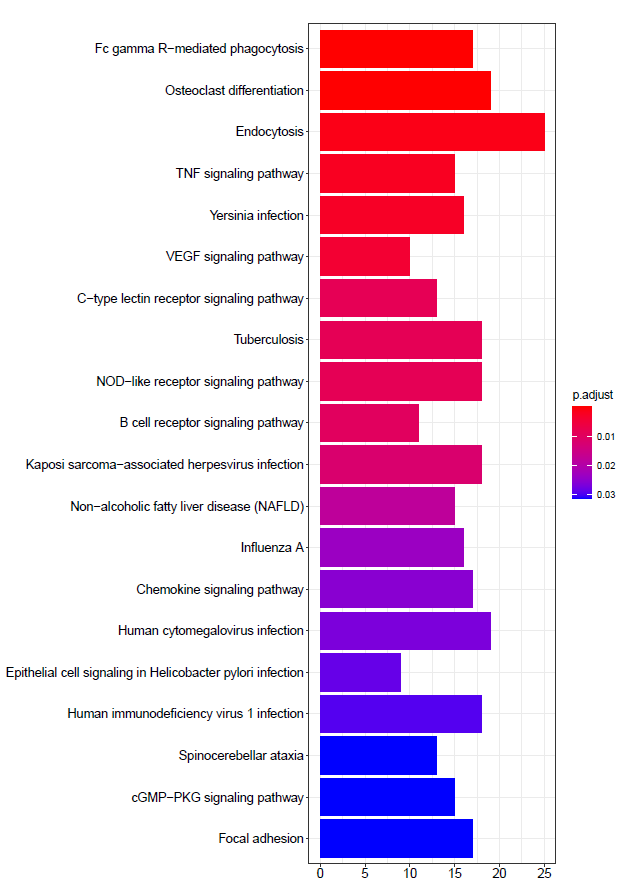


Fig. S2 KEGG pathway enrichment analysis of GDTLs.

Supplement: Supplementary file 2 — Supplementary Figure S2 [file 12020_2020_2523_MOESM2_ESM.docx]
